# Antibody Fc receptor CD16a mediates natural killer cell activation via mechanotransduction of piconewton forces

**DOI:** 10.1126/sciadv.aeb8946

**Published:** 2026-08-28

**Authors:** Rong Ma, K. Christopher Garcia, Bianxiao Cui, Markus W. Covert

**Affiliations:** ^1^ Department of Bioengineering, Stanford University, Stanford, CA 94305, USA.; ^2^ Department of Molecular and Cellular Physiology, Stanford University School of Medicine, Stanford, CA 94305, USA.; ^3^ Howard Hughes Medical Institute, Stanford University, Stanford, CA 94305, USA.; ^4^ Department of Chemistry, Stanford University, Stanford, CA 94305, USA.; ^5^ Wu Tsai Neurosciences Institute and ChEM-H Institute, Stanford University, Stanford, CA 94305, USA.

## Abstract

Natural killer (NK) cells eliminate target cells through antibody-dependent cell-mediated cytotoxicity (ADCC), initiated by CD16a (FcγRIIIa) recognizing the Fc region of antibodies bound to the target cell surface. While the recognition is considered to be driven by CD16a-Fc binding avidity, it fails to explain why Fc multimers inhibit ADCC in solution rather than trigger it. Here, we reveal that CD16a transduces piconewton forces and acts as a mechanosensor to facilitate NK activation. We demonstrate that CD16a force and the actin foci formation associated with it are essential for the phosphorylation of mechanosensitive adaptor Cas-L and signaling adaptor LAT (linker of activation of T cells), reshaping NK cell cytoskeletal dynamics and signaling. Our findings show that NK activation is an intricate process that integrates both biochemical and biophysical information and provide fresh mechanistic insight for immunoengineering.

## INTRODUCTION

**Fig. 1.**
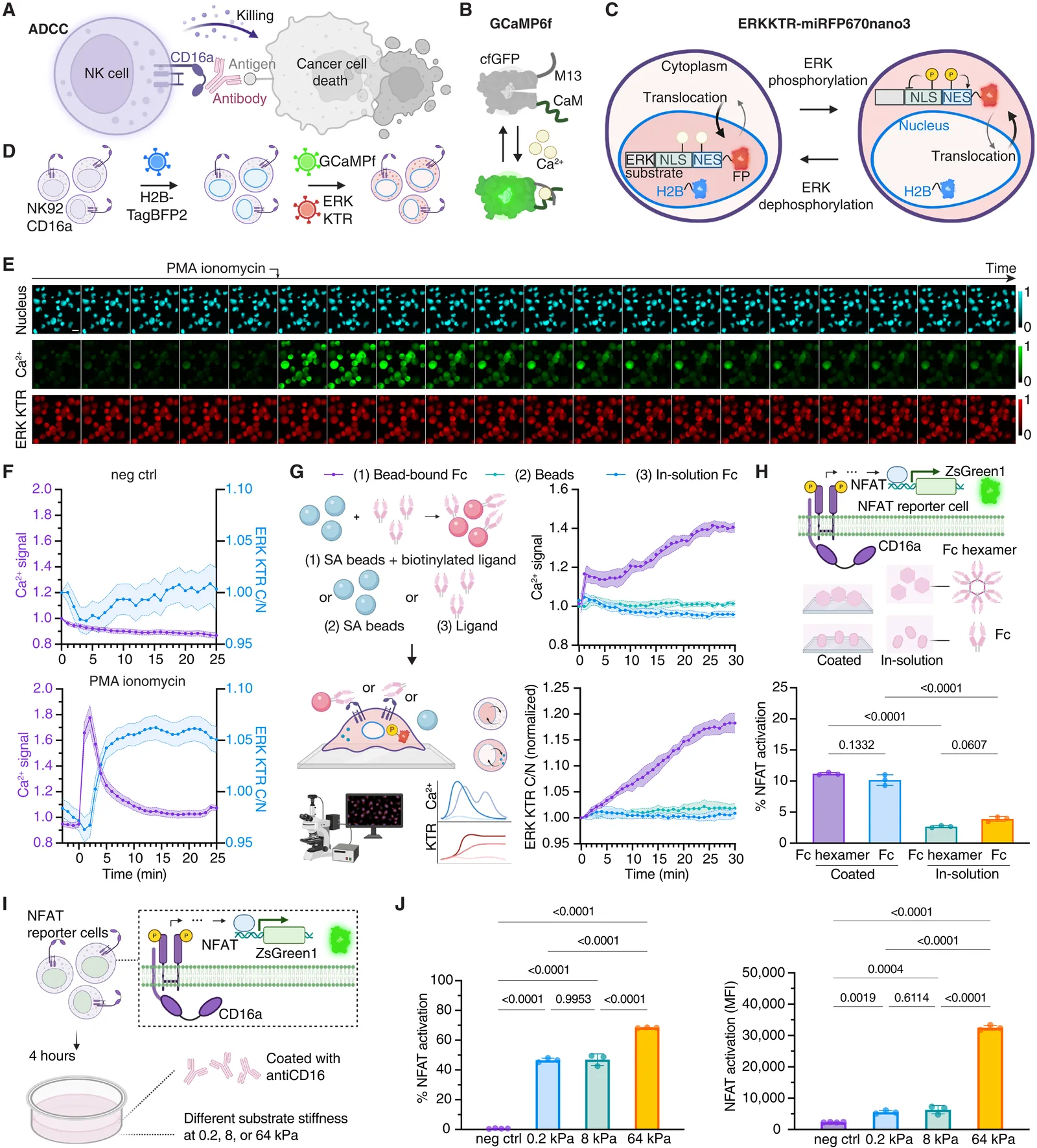
Mechanosensing contributes to potent NK cell activation by surface-bound IgG1 Fc. (**A**) Scheme illustrating ADCC mediated by CD16a in NK cells. (**B**) Scheme showing the GCaMP6f reporter used for monitoring Ca^2+^ flux. (**C**) Scheme showing the ERKKTR-miRFP670nano3 reporter used for monitoring ERK signaling. (**D**) Scheme illustrating the generation of NK92 CD16a reporter cell line by lentiviral transduction. (**E**) Representative images of reporter cells in H2B-TagBFP2 nucleus marker, Ca^2+^ signaling, and ERK signaling channels from *t* = −5 to 15 min upon PMA ionomycin stimulation. Scale bar, 20 μm; interval = 1 min. (**F**) Signaling activation of the entire population averaged from extracted single-cell traces. Data (means ± SEM) collected from three replicates with *n* = 173 cells for negative control (neg ctrl) and *n* = 233 cells for PMA ionomycin treatment. (**G**) Scheme and data showing that surface-bound Fc can trigger Ca^2+^ and ERK signaling potently compared to empty beads or Fc in solution in NK92 CD16a reporter cells. Data (means ± SEM) collected from three replicates, each replicate contains signaling traces of *n* = 300 to 400 cells per condition. Statistical analyses of dynamic signaling data are summarized in table S1. (**H**) Schematic and data show that either coated Fc or Fc hexamer can induce NFAT activation through CD16a, as compared to in-solution Fc or Fc hexamer (means ± SEM, data from three technical replicates, experiment was repeated twice). Statistical analysis was performed with unpaired two-tailed *t* test. (**I**) Schematic showing the measurement of NFAT activation on substrate coated with antiCD16. (**J**) Stiffer substrate can induce more NFAT activation in NFAT reporter cells; data (means ± SD) collected from three replicates. Statistical analysis was performed with one-way analysis of variance (ANOVA) and Tukey multiple comparison. MFI, median fluorescence intensity; SA, streptavidin.

Natural killer (NK) cells are lymphocytes in our innate immune system, protecting us from infections and cancer through their effector functions such as induced cytotoxicity and cytokine production. NK cell effector function is regulated by various receptor-ligand interactions at the cell-cell interface that are involved in target cell recognition. Following the discrimination of diseased cells from healthy cells, NK cells can kill target cells through a mechanism called antibody-dependent cell-mediated cytotoxicity (ADCC; Fig. 1A). ADCC is mediated by a fragment crystallizable (Fc) receptor, CD16a (FcγRIIIa), through a low-affinity interaction with the Fc region of an immunoglobulin G1 (IgG1) antibody molecule that is bound to an antigen on the target cell surface (fig. S1A) (*1*). ADCC is also one of the major strategies harnessed in cancer immunotherapy through antibody treatments, such as trastuzumab and rituximab.

However, our mechanistic understanding of ADCC is very limited. One question pertains to how NK cells can distinguish antibody molecules that are bound to a target cell surface from the ones that are freely circulating. Given the low affinity of CD16a-Fc binding (∼5 μM), sufficient avidity is often considered to be the primary factor in addition to affinity for driving ADCC (*2*). Although CD16a-Fc binding is not a multivalent interaction (1:1 stoichiometry), it is currently hypothesized in the field that cell-bound antibodies would potentially induce CD16a clustering in NK cells, which initiates signaling (*3*–*5*). CD16a microcluster formation and the subsequent centralization leads to the assembly of NK immune synapse and enables ADCC effector function. However, this hypothesis is insufficient in explaining NK activation: Engineered Fc multimers in solution can bind onto NK cells through multivalent binding and in principle bring several CD16a molecules together, evident by enhanced binding to cells using flow cytometry (*6*). Despite this enhanced avidity, these Fc multimers do not induce NK activation through ADCC without the Fab region facilitating immobilization to antigens on the target cell surface (*7*). On the contrary, ADCC can be inhibited by incubation with Fc multimers in solution, as was demonstrated with engineered Fc dimers, trimers, and hexamers (*6*, *8*–*10*). A recent report found that CD16a exist as dimers 17 nm apart in resting NK cells before activation, leading to more mysteries of NK activation through CD16a (*5*). The current clustering hypotheses cannot fully explain the mechanism, underlining a major gap in our fundamental understanding of NK cell activation.

In contrast to the less understood CD16a-mediated NK activation in innate immunity, the mechanism of its counterpart, T cell receptor (TCR)–mediated T cell activation in the adaptive immune system, is more explored (*11*, *12*). Parallel to CD16a, TCR triggering is the primary signal to induce effector function in T cells, which also results in the release of cytotoxic granules to kill cancer cells. Moreover, while TCR directly recognizes antigens on cancer cell surfaces whereas CD16a does this indirectly, both receptors signal through the association with ITAM (immunoreceptor tyrosine-based activation motif)–bearing adaptors such as CD3ζ and FcγR (*13*–*16*). Growing evidence has revealed that T cells leverage mechanical forces through TCR in the process of recognizing antigens, initiating signaling, and exerting cytotoxic function (*17*–*23*). Although there have been a few speculations of NK cells being mechanosensitive (*24*), these studies have been limited to adhesion receptor LFA-1 (*25*), pieozo-1 (*26*), and NKG2D (*27*–*29*). There was also one report that suggested CD16a could form catch or slip bonds with its nanobodies, but the study focused on measuring the binding kinetics of CD16a and its nanobody under flow in a cell-free system (*30*), lacking in-depth investigation of mechanical force transduction and its biological relevance.

Given the paralleled recognition and cytotoxic effector function of NK and T cells, we hypothesized that the primary immune activating receptor CD16a transmits mechanical forces and functions as a mechanosensor to dictate NK cell function. To test this hypothesis, we used microscopy approaches to investigate cell-generated mechanical behavior through CD16a in both engineered NK92 CD16a reporter cells and primary human NK cells. Specifically, we used high-throughput live cell imaging to capture the signaling dynamics in NK cells and molecular tension fluorescence microscopy (MTFM) to visualize CD16a mechanical activity. We show that CD16a transmits defined piconewton forces against its antibody and natural ligand IgG1 Fc during NK activation. The CD16a force generation and transmission is associated with actin foci dynamics, and the foci formation is almost perfectly colocalized to early total phosphorylation, as well as the phosphorylation of a key adaptor LAT (linker of activation of T cells) and a mechanosensitive scaffold protein Cas-L. Our findings suggest a mechanosignaling mechanism in NK cells during early activation.

## RESULTS

### Monitoring NK signaling dynamics with reporter cells

To study CD16a mechanosignaling, we first generated an NK92 reporter cell line to track the signaling dynamics in NK cells at single-cell level upon activation. NK92 cells do not naturally express CD16a, making them ideal for this study. We engineered NK92 cells to express low-affinity CD16a (F158) and confirmed the expression with flow cytometry (fig. S1B). To monitor NK signaling dynamics, we chose Ca^2+^ flux and extracellular signal–regulated kinase (ERK) phosphorylation as NK activation markers. We used GCaMP6f as the Ca^2+^ sensor (Fig. 1B), which consists of a calmodulin, a circularly permuted green fluorescent protein (cfGFP), and a peptide M13. The binding to Ca^2+^ leads to conformational changes in GCaMP6f and the modulation of solvent access and pKa of the chromophore, resulting in fluorescence increase (*31*). We used kinase translocation reporter (KTR) technology to obtain ERK phosphorylation dynamics (Fig. 1C). KTR is a fluorescent protein fused with a nuclear localization and exportation signal sequence (NLS and NES) and a specific kinase substrate for the kinase of interest. Depending on the target kinase activity, NLS and NES can get phosphorylated or dephosphorylated, leading to translocation into and out of the nucleus (*32*, *33*). This mechanism allows kinase activity information in individual cells to be obtained by extracting the cyto/nuclear fluorescence ratio from the translocated fluorescent protein, a far-red fluorescent protein miRFP670nano3. Therefore, we transduced the NK92 CD16a cells with lentivirus particles containing H2B-TagBFP2 to track cell nucleus, GCaMP6f to monitor Ca^2+^ flux, and ERKKTR-miRFP670nano3 to monitor ERK phosphorylation (Fig. 1D and fig. S1C). NK92 CD16a cells that expressed all constructs were isolated using fluorescence-activated cell sorting (FACS) to establish the NK92 CD16a reporter cell line (fig. S1D).

After generation of the NK92 CD16a reporter cell line, we tested whether these cells could report signaling dynamics by imaging them on positive and negative controls, which are poly-d-lysine (PDL)–coated plates with or without phorbol 12-myristate 13-acetate (PMA) ionomycin stimulation. PDL provides a nonspecific adhesion baseline, while PMA/ionomycin reliably induces robust Ca^2+^ influx and downstream ERK phosphorylation as a benchmark for reporter responsiveness. Cells were imaged with a 20× objective in a high-throughput manner so that the dynamic signaling of hundreds to thousands of cells can be monitored without biases. We adopted the previously reported image analysis pipeline CellTK for data extraction and analysis (fig. S2 and movie S1) (*33*). Representative images (Fig. 1E) and extracted activation traces of individual cells (fig. S3) show that both GCaMP6f and ERKKTR functioned as expected, with the fluorescence of GFP increased after PMA and ionomycin addition, and subsequently, the ERKKTR-miRFP670nano3 translocated from the nucleus to cytoplasm. By averaging the single-cell traces, we also obtained a population response, which showed that the calcium flux was triggered and peaked immediately, while ERK phosphorylation peaked at ∼5 min (Fig. 1F). We also confirmed that our reporter cell line is sensitive enough to respond to stimulations through CD16a at different antiCD16 coating concentrations (fig. S4).

### NK cells are activated when CD16a interacts with immobilized, but not soluble, ligands

After establishing the NK92 CD16a reporter cell system, we first confirmed that NK signaling is triggered by surface-bound CD16a ligands (Fig. 1G). We immobilized biotinylated human IgG1 Fc onto streptavidin beads and monitored NK cell signaling as bead-bound Fc was introduced. Empty streptavidin beads and the same amount of soluble IgG1 Fc were used as negative controls. We observed that both Ca^2+^ and ERK phosphorylation were triggered by bead-bound IgG1 Fc but not by soluble Fc or empty beads (Fig. 1G and fig. S5). We also confirmed this observation after Fc stimulation with an engineered Jurkat CD16a reporter cell line by measuring NFAT (nuclear factor of activated T cells) activity (fig. S6). Jurkat is an immortalized human T cell line that is frequently engineered as a standardized reporter platform for quantifying receptor-dependent activation. As this observation is often reasoned to be driven by avidity from multiple receptor-ligand binding events, we wondered whether increasing CD16a-Fc binding valency would result in significantly better activation in both soluble form and at cell-cell interface. To test whether high valency of soluble Fc can trigger CD16a signaling, we expressed a previously reported IgG1 Fc hexamer (fig. S7A) and examined its ability to induce NK cell activation (*34*). We found that it indeed cannot induce Ca^2+^ or ERK phosphorylation at 1 and 5 μg/ml (fig. S7B). However, when we coated IgG1 Fc hexamer and IgG1 Fc on glass, we were able to observe very similar level of activation by NFAT reporter cells (Fig. 1H and fig. S7C). To further examine the effect of valency at cell-cell interface, we engineered K562 cell line to express tethered IgG1 Fc, so that they can be used to directly stimulate NK cells without the need of antigen presentation and antibody incubation such as CD20 and rituximab (fig. S8, A and B). We found that tethered Fc dimer, despite potentially allowing two CD16a molecules to engage in proximity, offered higher Ca^2+^ triggering, but slightly less sustained ERK phosphorylation, and only a very minor increase (∼3%) in NFAT activation compared to cells incubated with the Fc monomer (fig. S8, C and D). Given that the Fc monomer and Fc dimer configurations differ in their tethering, we believe that other factors such as tether length and flexibility could have contributed to this result. Overall, our data indicate that NK cells can sense the bound or unbound state of the antibody interacting with CD16a, suggesting that the physical resistance exerted by the bound antibodies might play a role in NK activation.

### Substrate stiffness influences activation through CD16a

While induced CD16a clustering remains a possible contributor to NK activation through engaging surface-bound IgG1 Fc, the limited impact of Fc multivalency suggests that there could be other factors significantly driving this process. Therefore, we aimed to investigate whether the mechanism could be due to mechanosensing. To study mechanosensing at the cell-substrate interface, we coated substrates that have elastic moduli of 0.2, 8, or 64 kPa with antiCD16 (10 μg/ml) and then incubated the NFAT reporter cells on these substrates for 4 hours (Fig. 1I). We found that cells did not show changes in activation at 0.2 or 8 kPa; however, at 64 kPa, both NFAT activation levels and percent positive population were significantly higher (Fig. 1J). To also investigate at the cell-cell interface, after confirming that K562 IgG1 Fc target cells can induce potent signaling when coincubated with NK92 CD16a reporter cells (fig. S8B), we tracked signaling dynamics when NK cells interact with target cells that were treated with MβCD and MβCD-cholesterol. It was reported that MβCD can effectively increase target cell stiffness by ∼2-fold and MβCD-cholesterol can reduce target cell stiffness by ∼40% in multiple adherent cell lines, measured by atomic force microscopy, optical tweezers, as well as deformation cytometry (*35*). Although K562 is a nonadherent lymphoblast cell line, we adopted this method to explore whether there would be differences in activation. We treated K562 IgG1 Fc cells with MβCD to remove cholesterol from the cell membrane, or with MβCD-cholesterol to load more cholesterol into the cell membrane. The cholesterol removal and loading were confirmed by Filipin III staining (fig. S9, A and B), and the treated K562 IgG1 Fc cells were subsequently introduced to NK92 CD16a reporter cells. We found that the cells treated with MβCD can trigger Ca^2+^ flux and ERK phosphorylation more potently than the ones treated with MβCD-cholesterol (fig. S9, C and D, and movie S2). Together, these experiments indicate that NK cell activation may require a finely regulated mechanosensing machinery.

### CD16a transmits defined piconewton forces and acts as a mechanosensor in NK cells

**Fig. 2.**
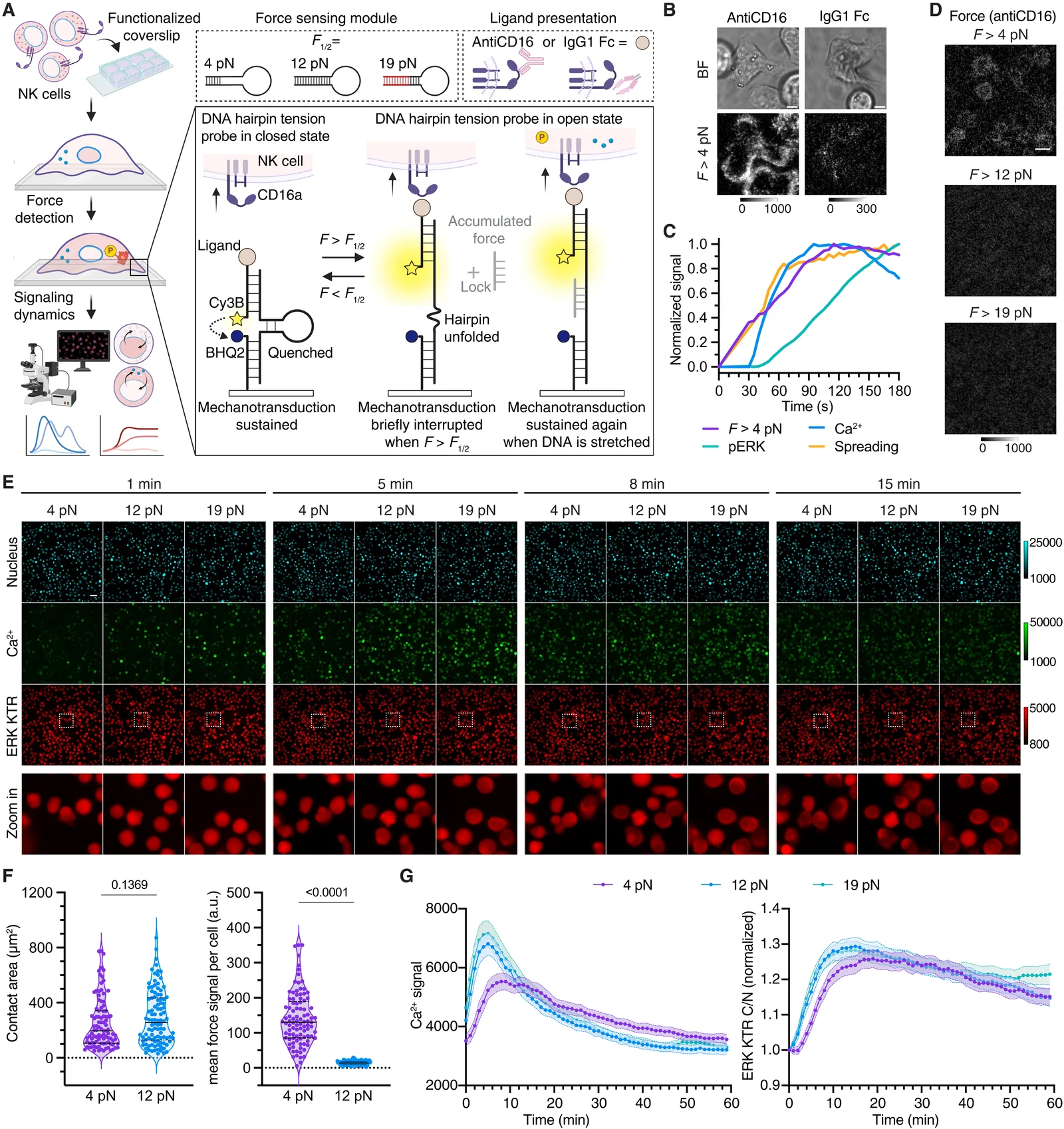
CD16a transmits defined piconewton forces and acts as a mechanosensor in NK92 cells. (**A**) Schematic showing the measurement of CD16a forces and signaling dynamics using DNA tension probes and NK92 CD16a reporter cells. (**B**) Representative 100× bright-field and fluorescence images of CD16a forces against antiCD16 and IgG1 Fc. Scale bars, 5 μm. (**C**) Representative traces of sequential events during NK activation on antiCD16 DNA tension probe substrate in a representative NK92 CD16a reporter cell, including cell spreading, CD16a forces greater than 4 pN, Ca^2+^ triggering, and pERK signaling. Experiment was repeated three times. (**D**) Representative 20× fluorescence images showing NK92 CD16a reporter cells producing forces greater than 4 pN but less than 12 and 19 pN against antiCD16. Scale bar, 20 μm. (**E**) Representative fluorescent images of cell nucleus, Ca^2+^ flux, and ERKKTR translocation (with zoom in) during NK92 CD16a reporter cell activation on DNA tension probes presenting antiCD16 at different time points. Scale bar, 50 μm. (**F**) Contact area and mean force signal per cell of NK92 CD16a cells incubated on antiCD16 DNA tension probe substrates at *t* = 10 min. Experiment was performed in three replicates, *n* = 97 and 102 cells. Statistical analysis was performed with unpaired two-tailed *t* test. (**G**) Population response of NK92 CD16a reporter cells incubated on different DNA hairpin probes. Experiment was performed in three replicates, data averaged (means ± SEM) from single cell traces of Ca^2+^ triggering and ERK phosphorylation, *n* = 396 to 669 cells per condition. Statistical analyses of dynamic signaling data are summarized in table S1. a.u., arbitrary unit.

We next sought to dissect the NK mechanosensing behavior through CD16a in a quantitative manner by coupling high-throughput imaging of cell signaling dynamics with MTFM. DNA hairpin tension probes are a superior MTFM tool for receptor force studies, as they provide the highest spatiotemporal resolution as well as force sensitivity (*20*, *36*). DNA hairpin tension probes use fluorophore-quencher pairs to report on the mechanical extension and unfolding of DNA hairpins under force (Fig. 2A, closed and open state of DNA hairpin tension probe). The hairpin is hybridized to a fluorescent (Cy3B) ligand strand on one arm and a quencher (BHQ2) anchor strand on the other arm, and subsequently immobilized on a glass substrate (fig. S10A). In the absence of mechanical force, the hairpin is closed, and thus, the fluorescence is quenched; in the presence of a mechanical force applied to the ligand presented on top of the probe that is greater than the hairpin *F*_1/2_ (the force at equilibrium that leads to a 50% probability of unfolding), the hairpin mechanically unfolds and generates a fluorescent signal. This digital probe design is highly modular, which enables mapping forces above different magnitudes simply by using a hairpin with a different *F*_1/2_ (Fig. 2A, force sensing module). We presented both antiCD16 and IgG1 Fc to NK92 CD16a cells to evaluate their mechanical force profile (Fig. 2A, ligand presentation).

We prepared substrates functionalized with 4, 12, and 19 pN DNA tension probes at the same density (fig. S10, B to D), and to ensure that we could pick up the potential transient and weak forces, we also used a previously reported lock oligonucleotide to amplify the tension signal over time by mechanically selective hybridization (fig. S11A) (*19*). We imaged NK cells on the tension probe substrates presenting antiCD16 or IgG1 Fc, while substrates with no ligand served as a negative control (fig. S11B). We found that NK92 cells were able to generate and transmit mechanical forces greater than 4 pN through CD16a during activation. The observed CD16a force against antiCD16 exhibited a puncta pattern during cell spreading and was also located at the periphery of the cell (Fig. 2B, antiCD16). While CD16a forces greater than 4 pN against antiCD16 were consistently observed in NK92 cells, only a fraction of cells showed forces greater than 4 pN against IgG1 Fc, and in those that did, force signals were less abundant (Fig. 2B, IgG1 Fc, and movie S3). This observation means that force transmission still exists through natural receptor-ligand binding; however, it is typically too weak to unfold the 4 pN hairpins. Due to the difficulties in force visualization against the natural ligand Fc in NK92 CD16a reporter cells, as well as obtaining strong population-level dynamic signaling data (fig. S11C), we decided to focus on using antiCD16 to interrogate the mechanosensing mechanism through CD16a in NK92. Using CD16a antibody also has significant physiological relevance, as CD16a antibody or single-chain variable fragments (scFVs) are often used in bispecific or trispecific engager designs that aim to recruit NK cells for their cytotoxicity function (fig. S1A) (*37*–*39*). We consistently observed that after the cell first contacted ligand-presenting substrates, CD16a force generation accompanied cell spreading at a similar rate, followed by Ca^2+^ triggering and then ERK phosphorylation (Fig. 2C, fig. S12, and movie S4). The forces NK92 cells generated through individual CD16a against antiCD16 were mostly between 4 and 12 pN, as much less hairpin opening was observed with the 12 pN probe and 19 pN probe (Fig. 2D). With high-throughput imaging of signaling dynamics, we noticed that, although the DNA hairpin substrates at different *F*_1/2_ were almost identical chemically, the rate of NK activation at the population level was different. Representative images (Fig. 2E and movie S5) show that at 1 min, fewer cells on 4 pN probe exhibited Ca^2+^ triggering than cells seeded on 12 and 19 pN probes. At 5 min, although cells on all substrates showed Ca^2+^ signal increase, cells on 12 and 19 pN substrates had a stronger response accompanied with ERKKTR translocation. By 8 min, cells on all substrates showed a similar level of Ca^2+^, while there were still more cells on 12 and 19 pN substrates with ERKKTR translocation. Later at 15 min, the Ca^2+^ and ERK activity looks identical for all three substrates. Quantitative analysis of cell spreading area and force signal (Fig. 2F), as well as the extracted signaling traces (Fig. 2G), indicates that the hairpin opening slightly negatively affected signaling, evident by a slower onset and smaller peak in Ca^2+^ signaling, as well as a slower response in ERK phosphorylation. We suspect that this observation was due to brief interruption of mechanotransduction with 4 pN hairpin unfolding (Fig. 2A, mechanotransduction) and further supports the significant role of CD16a mechanosensing in NK activation. Overall, we combined live-cell imaging of receptor forces and NK intracellular signaling to quantify the mechanical forces at the piconewton scale at which NK are activated by CD16a.

### Actin dynamics and foci formation contribute to CD16a mechanics and NK activation

**Fig. 3.**
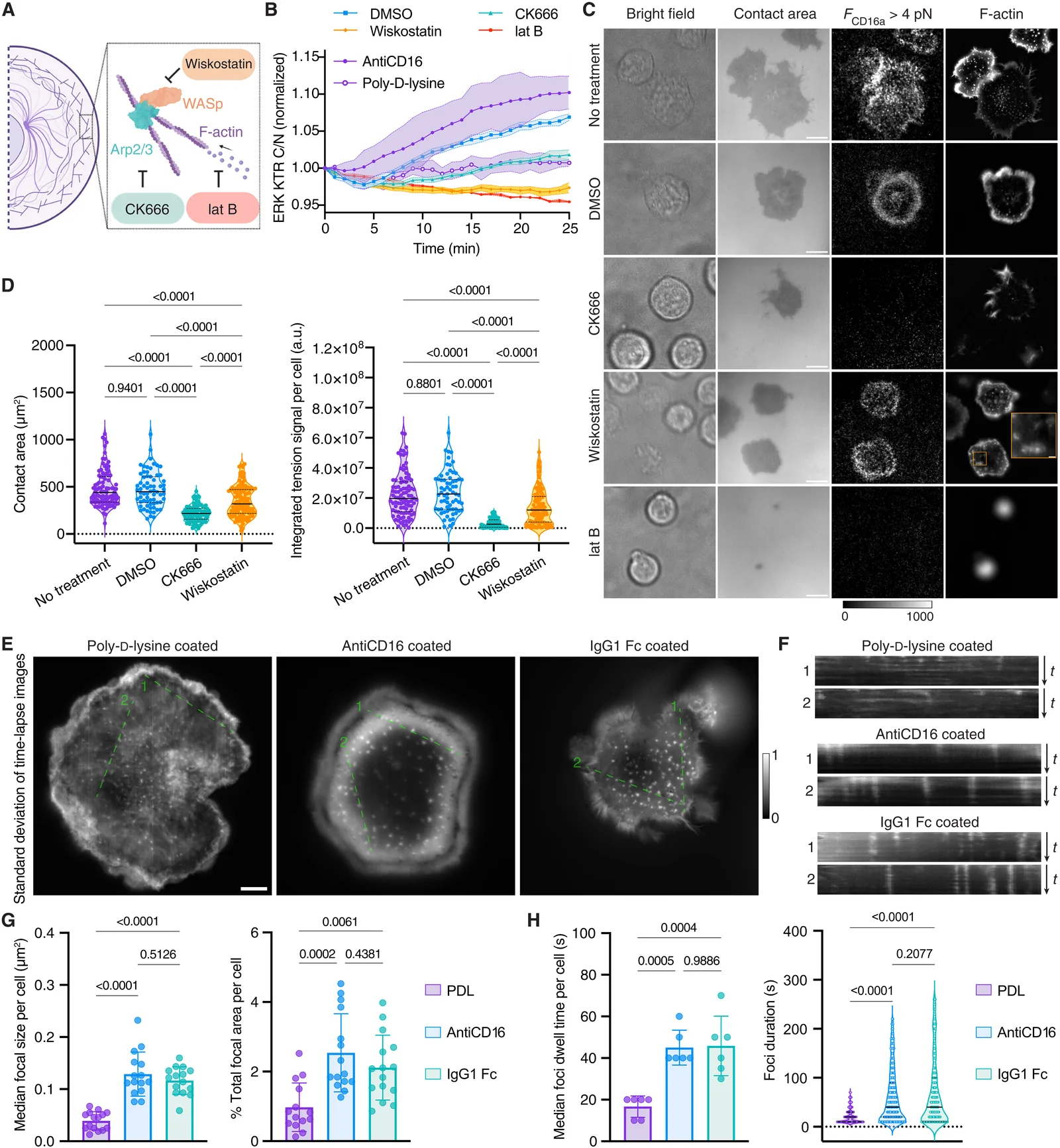
Actin dynamics affect CD16a forces and are involved in NK activation. (**A**) Schematic showing the inhibition of specific cytoskeletal activities. (**B**) Extracted ERK traces show that NK activation is inhibited with CK666, wiskostatin, and lat B; means ± SEM from three replicates; each condition contains traces of *n* ≈ 200 cells per replicate. Statistical analyses of dynamic signaling data are summarized in table S1. (**C**) Representative bright field, contact area, tension and actin images of NK92 CD16a cells under different treatment. Scale bars, 5 μm. In the wiskostatin group, a zoom-in view of F-actin is shown in the orange box. Scale bar, 1 μm. (**D**) Quantitative analysis on contact area and forces. Data were collected from cells in three replicates, *n* = 95, 68, 100, 122 cells for the respective treatments. Solid line indicates the median, dashed line indicates the quartiles, and statistical analysis was performed with one-way ANOVA and Tukey’s multiple comparison. (**E**) Standard deviation images of F-actin time-lapse in representative cells from *t* = 5 to 10 min, interval = 10 s. Scale bar, 5 μm. (**F**) Kymographs of ROIs in representative cells marked in (E). (**G**) Median focal size per cell and the percentage of total focal area occupied of the cell contact area at *t* = 5 min. Data were analyzed from representative cells, three replicates. Means ± SD, *n* = 15 cells per condition, foci count ranges from 23 to 290 per cell. Statistical analysis was performed with one-way ANOVA and Tukey’s multiple comparison. (**H**) Dwell time of actin foci for cells incubated on PDL, antiCD16, and IgG1 Fc substrates from *t* = 5 to 10 min, means ± SD. Measurements were taken with 10-s intervals, and dwell time was obtained by particle tracking analysis. Statistical analysis was performed with one-way ANOVA and Tukey’s multiple comparison. Data were collected from *n* = 6 representative cells and foci counts = 544, 616, 378 in three replicates.

As we identified CD16a as a mechanically active receptor, we next aimed to elucidate cellular input that could contribute to the CD16a mechanical activity. We chose small-molecule inhibitors CK666, wiskostatin, and latrunculin B (lat B) to pretreat NK92 CD16a reporter cells for 20 min to inhibit Arp2/3 in actin branching, WASp/N-WASp regulation of cytoskeleton (*40*), as well as actin polymerization prior to incubating cells on PDL or antiCD16-coated wells (Fig. 3A). Compared to cells on antiCD16 without treatment or treated with only dimethyl sulfoxide (DMSO), CK666, wiskostatin, and lat B abolished ERK phosphorylation in NK activation through CD16a (Fig. 3B). This inhibition of activation was also confirmed in NFAT signaling using Jurkat CD16a NFAT reporter cells (fig. S13).

We then examined the effect of inhibiting cytoskeletal activities on NK cell spreading and CD16a force transmission using 4 pN DNA tension probes presenting antiCD16. The inhibition of Arp2/3 by CK666 and WASp/N-WASp by wiskostatin reduced NK spreading area and dramatically decreased observed forces through CD16a (Fig. 3, C and D). Cells treated with lat B did not spread or produce CD16a forces, and thus, we did not include it in quantitative analysis. To obtain more mechanistic insight, we engineered NK92 CD16a cells to express an F-actin marker LifeAct-mNeonGreen for these experiments (fig. S14). Although some cell spreading was maintained upon CK666 treatment, we found that the formation of actin foci was eliminated (Fig. 3C, F-actin). For cells treated with wiskostatin, F-actin concentrated in small patches and showed some only semiformed actin foci (Fig. 3C, wiskostatin). Based on this evidence, we suspect that actin foci play a role among the cytoskeletal activities that are important for NK activation.

To further investigate actin foci formation, we followed their dynamics on activating ligand-coated glass compared to nonactivating PDL-coated glass. The standard deviation of time-lapse images (Fig. 3E and movie S6) of NK92 CD16a LifeAct cells showed the most dynamic F-actin changes during image acquisition, from which we found that actin foci were more pronounced on activating substrates than PDL substrate. Kymographs of actin foci [region of interest (ROI) 1 and 2 marked, green dash in Fig. 3E] showed that actin foci on activating ligands were more stable (Fig. 3F). Quantitatively, on antiCD16 and IgG1 Fc substrates, there was no significant difference in size of actin foci and percent area actin foci occupied of total contact area per cell, while both substrates yielded significantly larger actin foci than cells spreading on PDL (Fig. 3G and fig. S15). Particle tracking analysis by TrackMate (Fig. 3H and movie S7) showed that the median dwell time of individual actin foci was ∼40 to 50 s on antiCD16 and IgG1 Fc substrates, significantly longer than that on PDL substrate. In summary, we found that actin cytoskeletal activities contribute significantly to NK cell activation, in part due to the formation of relatively larger actin foci that have a longer dwell time when engaging activating ligands on the tested planar surfaces.

### CD16a force-associated actin foci are phosphorylation sites for the adaptor proteins Cas-L/LAT

**Fig. 4.**
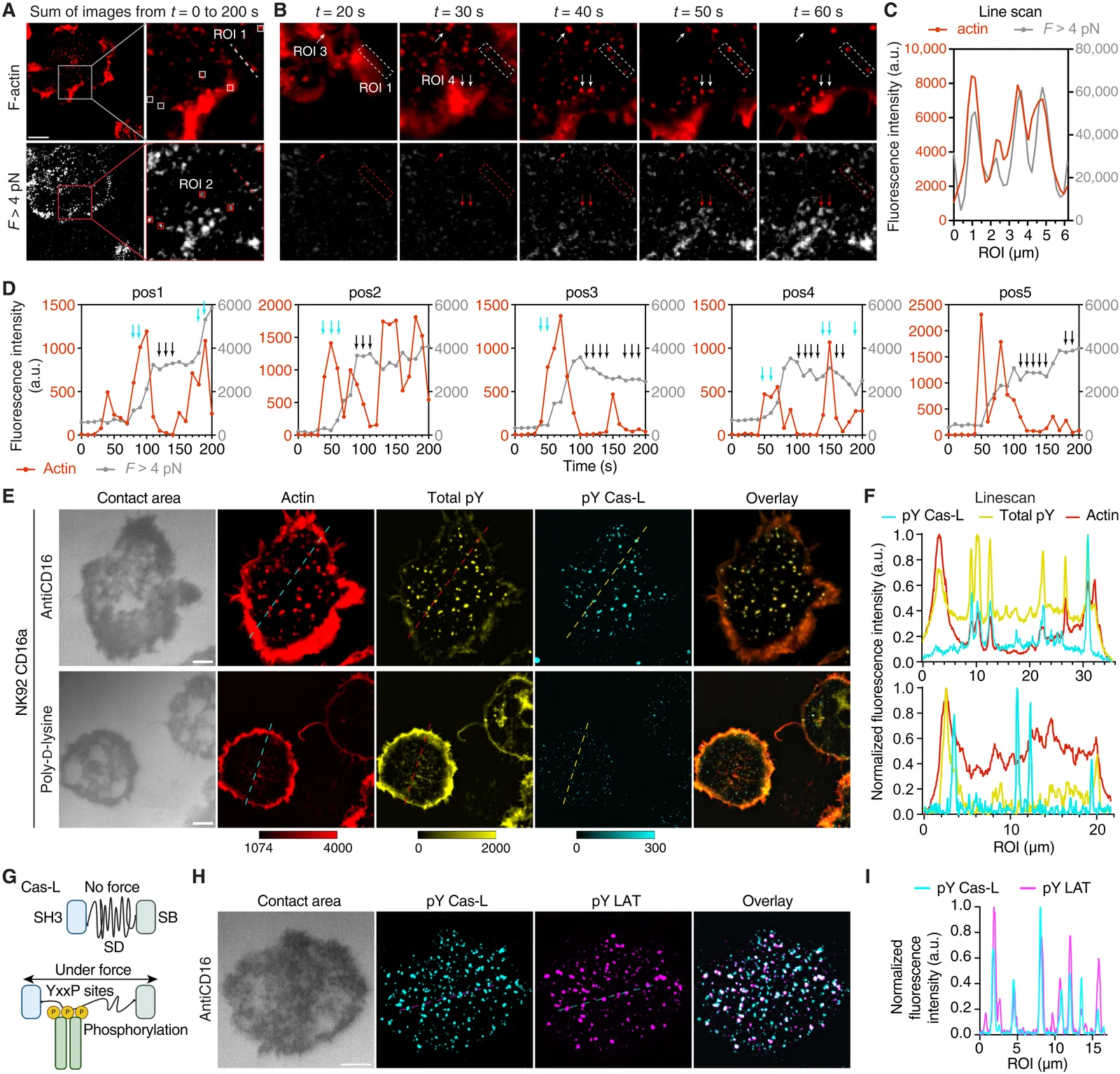
CD16a force is associated with actin foci and sites of signaling adaptor phosphorylation. (**A**) Sum of time-lapse F-actin and accumulated force images from *t* = 0 to 200 s from a representative cell. Column on the right is a zoom-in view of the box with two ROIs, ROI 1 is a line scan and ROI 2 are five 4 by 4 pixel boxes. Scale bar, 5 μm. (**B**) Time-lapse F-actin and accumulated tension images of the zoom-in view with two more ROIs marked by arrows. (**C**) Line scan of ROI 1 in sum images showing the colocalization between F-actin and tension channels. (**D**) Time course plots of F-actin and accumulated tension signal for ROI 2. Cyan arrows mark the actin foci assembly followed by accumulated force signal increase, while black arrows mark the disassembly of actin foci followed by accumulated force signal plateauing. (**E**) Representative immunofluorescence staining images of actin, total pY, and pY Cas-L in NK92 CD16a cells incubated on antiCD16 or PDL substrates at *t* = 10 min. Scale bar, 5 μm. (**F**) Line scan of the dashed line ROI marked in (E) showing the colocalization of actin foci, total pY, and pY Cas-L. Fluorescence intensity in each channel was normalized. (**G**) Scheme illustrating mechanosensing mechanism by Cas-L. (**H**) Representative immunofluorescence staining images of pY Cas-L and pY LAT in NK92 CD16a cells incubated on antiCD16 substrate at *t* = 10 min. Scale bar, 5 μm. (**I**) line scan of the dashed line ROI marked in (H) showing the colocalization of pY Cas-L and pY LAT. Fluorescence intensity in each channel was normalized.

Based on these observations, we next sought to explore the connection between actin foci, CD16a force, and NK activation. We imaged accumulated CD16a forces using NK92 CD16a cells expressing LifeAct-mNeonGreen as they spread and exert force on 4 pN DNA hairpin probes presenting antiCD16 with the lock oligonucleotide (movie S8). We found that the puncta pattern in CD16a forces resembled the actin foci formation. To find their association patterns, we summed the images from the first 200 s in the F-actin channel as well as the tension channel (Fig. 4A). Images were marked with ROI 1 (a line scan) and ROI 2 (five small squares) in the F-actin channel and in the tension channel. Figure 4B shows images of F-actin and tension of the same region from 20 to 60 s, with two more ROIs (ROI 3 and 4, arrows) labeled on the images. In the ROI 1 line scan on the summed image, we found that actin foci colocalized with sites of accumulated CD16a force signal (Fig. 4C). As observed in ROI 3 and 4 in Fig. 4B, we noticed that actin foci were assembled first prior to CD16a force transmission exceeding 4 pN. Based on this, we tracked the intensity of the randomly picked positions on sum images (ROI 2) and observed that actin assembly frequently took place 10 to 20 s prior to the transmission of CD16a forces that were greater than 4 pN (Fig. 4D, cyan arrows). Similarly, with a bit of lag, actin disassembly was often associated with no new CD16a force transmission, leading to a plateau in the force plot (Fig. 4D, black arrows).

While it was exciting to establish the connection between actin foci and CD16a forces, we were curious about the signaling function of these sites of force-foci association. After incubation on substrates coated with either antiCD16 or PDL (negative control), NK92 CD16a cells were fixed and stained for F-actin and total phosphorylation (Fig. 4E). We found that during early NK cell activation on the antiCD16 surface, actin foci were colocalized with sites of total phosphorylation (Fig. 4F, top). Poor colocalization was observed with cells incubated on the PDL surface (Fig. 4F, bottom). Since actin foci are associated to sites of CD16a force transmission and total phosphorylation, we next wondered whether any specific intracellular protein could be involved in the CD16a mechanotransduction. P130Cas is one of the mechanosensing adaptors involved in cell adhesion, and it has been found that the phosphorylation YxxP sites in the substrate domain are only exposed if the protein is stretched (*41*). It is shown that the most abundant member of Cas family proteins in lymphocytes is Cas-L (Fig. 4G), which can affect T cell signaling and activation (*42*–*44*). We stained for the phosphorylation of Cas-L and found that it also colocalized to actin foci and total pY (Fig. 4, E and F), with the pY Cas-L and total pY Pearson’s correlation coefficient = 0.81 ± 0.06 (mean ± SEM). To further establish the mechanotransduction mechanism between CD16a force, actin foci, and phosphorylation on Cas-L, we sought to determine whether any signaling adaptor could be involved. Thus, we stained for the phosphorylation of LAT, which is a key adaptor protein in immune cell signaling and known to be responsive upon Fc receptor stimulation (*45*). We found that the phosphorylation of LAT also colocalized with phosphorylation of Cas-L (Pearson’s correlation coefficient = 0.52 ± 0.03, mean ± SEM), especially to the larger patches (Fig. 4, H and I). Therefore, we concluded that mechanotransduction through Cas-L likely contributes to signal propagation via CD16a mechanosensing during NK activation.

### CD16a transmits higher magnitude of forces for mechanosensing in primary NK cells

**Fig. 5.**
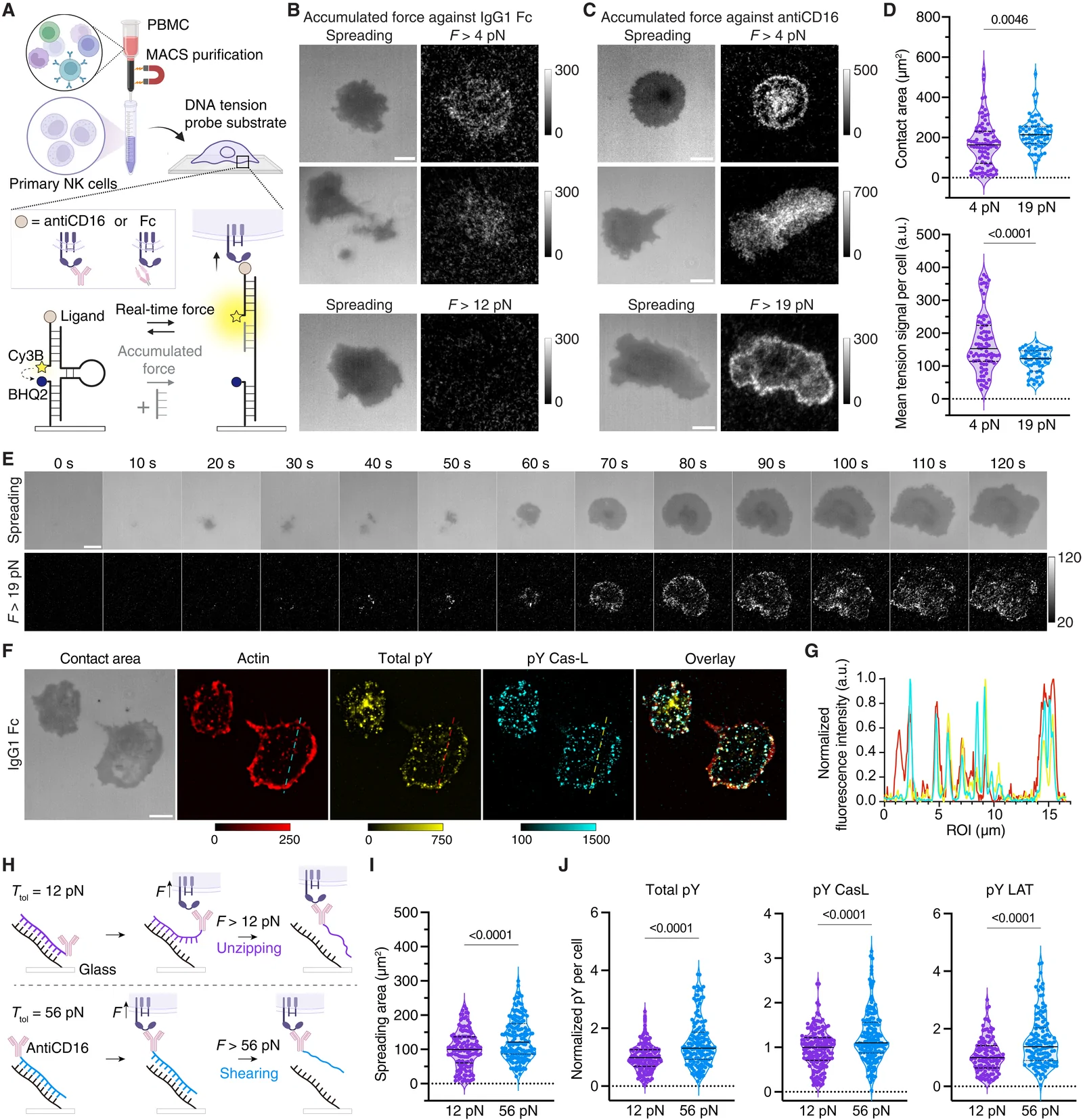
CD16a transmits piconewton forces and senses different mechanical environments in primary NK cells. (**A**) Schematic shows the MACS purification of primary NK cells from PBMC and force measurements on DNA tension probe substrates in real-time or accumulated manner (shown in gray). (**B**) Representative images showing CD16a forces generated by primary human NK cells against IgG1 Fc were greater than 4 pN but smaller than 12 pN. Scale bar, 5 μm. (**C**) Representative images showing CD16a forces generated by primary human NK cells against antiCD16 were greater than 19 pN. Scale bars, 5 μm. (**D**) Quantitative analysis shows the contact area and accumulated CD16a force against antiCD16 in primary NK cells. Data were collected from three replicates, *n* = 83 and 66 cells; solid line represents median and dashed lines represent quartiles. (**E**) Time-lapse images of a representative primary NK cell spreading and exerting real-time force greater than 19 pN against antiCD16. Scale bar, 5 μm. (**F**) Representative immunofluorescence staining images of actin, total pY, and pY Cas-L in primary NK cells incubated on IgG1 Fc substrates at *t* = 10 min. Scale bar, 5 μm. (**G**) Line scan of the dashed line ROI marked in (F) showing the colocalization of actin foci, total pY, and pY Cas-L. Fluorescence intensity in each channel was normalized. (**H**) Schematic showing TGTs as tools to manipulate the force threshold allowed for mechanotransduction. As soon as the TGT ruptures, the mechanotransduction is terminated. (**I**) Spreading area and (**J**) total, Cas-L, and LAT phosphorylation in primary NK cells on TGT presenting antiCD16 at *t* = 5 min. Means ± SD; data collected from 3 to 4 replicates; *n* ≈ 115 to 199 cells per condition; solid line indicates median and dashed line indicates quartiles.

Last, we confirmed the mechanical activity of CD16a in primary NK cells. We purified primary human NK cells from peripheral blood mononuclear cells (PBMCs) and imaged the cells using substrates functionalized with DNA hairpin tension probes (Fig. 5A). Primary NK cells can indeed exert defined forces through CD16a to IgG1 Fc compared to negative control (fig. S16), and we consistently observed accumulated forces against IgG1 Fc (movie S9), in between 4 and 12 pN (Fig. 5B). Unexpectedly, CD16a forces against antiCD16 in primary NK cells were higher than that in NK92 cell line (Fig. 5C and movie S10). Quantitative analysis also showed that cell spreading was affected by different hairpin mechanical threshold, with accumulated forces greater than 19 pN against antiCD16 (Fig. 5D). Time-lapse imaging of real-time forces show that as soon as the cell made an initial contact with ligands, force was immediately transmitted through CD16a with a puncta pattern (Fig. 5E, 10 to 30 s) and then expanded to the periphery of the cell as the cell spread (70 to 120 s), consistent with what we see in NK92 CD16a cells (movie S8).

We also confirmed that actin foci still colocalized to total phosphorylation and phosphorylation of the mechanosensing Cas-L protein in primary NK cells (Fig. 5, F and G), which possibly facilitated force transmission together. We then verified CD16a mechanosensing mechanism using the tension gauge tethers (TGTs) to present antiCD16, because TGTs can provide a higher force threshold that can withstand the greater than 19 pN forces that CD16a transmits to antiCD16 in primary NK cells. TGTs are chemically identical DNA duplexes that can be mechanically separated in unzipping or shearing geometry, leading to duplex rupture at either 12 or 56 pN and subsequent termination of mechanotransduction through CD16a (Fig. 5H). We found that primary NK cells exhibited larger contact area (Fig. 5I) and higher total phosphorylation, Cas-L phosphorylation, and LAT phosphorylation on 56 pN TGT rather than 12 pN (Fig. 5J). This is likely a result of more sustained mechanotransduction on 56 pN TGT, as CD16a forces in primary NK cells are above 12 and even 19 pN against antiCD16 and thus can easily rupture the 12 pN TGT and terminate CD16a mechanotransduction. The difference of phosphorylation on 56 and 12 pN TGTs further illustrates the mechanosensing ability of primary NK cells, which echoes the observations in NK92 CD16a reporter cells, although the force magnitude and mechanosensing thresholds differed (Fig. 2G). These differences could be dependent on the intrinsic biophysical properties and cytoskeletal dynamics of NK92 cells and primary NK cells.

## DISCUSSION

Surface-bound ligands can induce potent immune responses in both the innate and adaptive immune system. Examples include activation through TCR in T cells, B cell receptor (BCR) in B cells, and Fc receptor in NK cells. One widely accepted activation mechanism is that the binding to ligands can induce receptor clustering, which increases the local density of ITAMs and thereby increases the rate of phosphorylation on kinases and adaptors for signaling (*13*, *46*). However, clustering cannot explain activation sufficiently. For T cell activation, a recent study showed that TCR engaging a single pMHC was enough for triggering the activation signal (*47*). In the case of Fc receptor–mediated activation, Fc multimers lacking antigen binding abilities but with enhanced binding to Fc receptors can actually inhibit antibody-mediated effector functions including cytotoxicity (ADCC) and phagocytosis (ADCP) and thus are used as therapeutic strategies for autoimmune conditions. Together, these observations suggested that there could be other driving forces behind activation through the Fc receptor CD16a.

NK cells and CD16a’s counterparts, T cell and TCR triggering has been investigated extensively at both the molecular and cellular level, with significant findings emerging from a variety of perspectives, for instance, models of kinetic proofreading, mechanosensing, phase separation, and size exclusion (*48*–*51*). These models greatly contributed to explaining the activation mechanism through TCR. However, to date, NK activation lacks such extensive investigation, especially with newer technologies. As an important process that drives ADCC, which is harnessed in many therapeutical antibodies, the insufficient mechanistic understanding greatly limits our ability to design immunotherapies that recruit NK cells more efficiently.

This work fills the gap by interrogating NK activation process via primary activating receptor CD16a from a biophysical perspective. We found that CD16a is a mechanically active receptor, capable of exerting forces greater than 4 pN to IgG1 Fc during NK activation. Marrying signaling reporters and MTFM, we were able to track the force events and dynamic signaling at the same time. As mechanical engagement of receptor ligand propagated, the Ca^2+^ flux was triggered and followed by sustained ERK signaling. One important observation was that when cells first encountered ligands on 4 pN hairpin tension probe, it took longer for them to trigger relative to higher threshold probes. We reason that the cells can unfold the 4 pN hairpins and thus do not sense the reaction from immobilized ligand until the unfolded hairpin is fully stretched, which led to a slower activation after exerting a force through CD16a at the population level.

Given that small-molecule inhibition of cytoskeleton impaired both CD16a forces and NK activation, we suspected that actin foci, a pattern specific to activating substrates driven by Arp2/3, are involved in NK mechanosensing ability through CD16a. Actin foci have been observed in immune cells incubated on two-dimensional (2D) planar substrates. Studies at cell-substrate interface have revealed that actin foci are leveraged by B cells to extract antigens (*52*) and that they are important in T cell activation and lytic immune synapse formation (*53*, *54*). It is also reported that actin foci colocalize to TCR microclusters during T cell activation (*55*) and that the Arp2/3 facilitated fast F-actin turnover rate that likely contributes to the fast response from T cells (*54*, *56*). These studies prompted us to investigate the connection between cytoskeleton, CD16a force, and NK mechanosensing. Consistent with the studies in T cells and B cells, we also found relatively large actin foci formed on both antiCD16 and IgG1 Fc substrates with an average dwell time around 50 s, which we suspect to be likely involved in mechanotransduction and signal propagation. We were able to identify actin foci as part of the sites of where CD16a mechanotransduction took place, evident by the colocalization to the sites of total phosphorylation including Cas-L. Due to the nature of how Cas-L has to be stretched on both ends to reveal the phosphorylation sites, our finding elucidated a potential mechanosensing network. Although we were able to observe that force transmission through CD16a follows actin foci assembly, we currently have not yet determined whether the CD16a force transmission occurs before or after Cas-L phosphorylation. Thus, an important remaining question is whether the phosphorylation on Cas-L scaffold protein is a necessary condition for relaying CD16a force or if it is a response from CD16a mechanosensing and NK activation signal propagation. Answering this question is not only helpful in understanding NK activation, but can also provide insight in other immune cell triggering mechanisms.

Following our findings in the NK92 cell line, we further validated our results in primary human NK cells. CD16a indeed can transmit forces; however, the force was greater than 19 pN to antiCD16 and only greater than 4pN to IgG1 Fc. These data suggest that CD16a-antiCD16a can withstand mechanical forces of at least 19 pN. Moreover, this indicates that the lack of consistent 12 pN force signal through the NK92 CD16a-antiCD16a interaction is because NK92 cells pull with weaker force and cannot open the 12 pN hairpins efficiently. The differential force magnitude raises another interesting question: What contributes to the force generation and sensing differences between primary NK cells and NK92 cell line? While CD16a polymorphism (158 F/V) could contribute to the differences in force profile due to their differential ability to bind to IgG1 Fc (*57*), the binding strength between NK92 and primary NK cells with the pan-antiCD16 clone 3G8 should not be affected. If immune receptor mechanosensing machinery is a pure intrinsic property of the molecular interaction interface, we should see similar force profile in NK92 and primary NK cells. Consistent with our force measurements in primary NK, we also observed differences in activation level by phosphorylation at a higher threshold force of 56 pN compared to 12 pN, supporting our finding of NK mechanosensing through CD16a.

Mechanistically, while there are paralleled processes in T cells through TCR and NK cells through CD16a, there are also plenty of differences in these two systems. First, CD16a is regulated by ADAM17, which results in CD16a shedding upon NK activation (*58*). Whether ADAM17 could regulate NK mechanosensing abilities and potentially lead to NK cell fate of expansion or exhaustion is unknown. Second, unlike TCRs that are polyclonal, CD16a has limited polymorphism, which could contribute to differential mechanosensing abilities. For example, one of the most known variants is CD16a F158V, which has a higher affinity to IgG1 Fc and found in a smaller percent of the population (*59*). This difference in affinity also raised a third critical question in this comparison: In T cell mechanosensing, the low-affinity TCR-pMHC interactions often exhibit a distinct catch-bond behavior, which is thought to minimize the interference by nonspecific antigens and amplify signals specific to cognate antigens (*18*, *21*–*23*, *60*, *61*). As a low-affinity interaction, does CD16a-Fc exhibit catch-bond behavior? Fourth, Fc glycosylation is known to affect the binding between Fc and Fc receptors, and engineered Fc variants can achieve enhanced ADCC and ADCP by afucosylation (*62*, *63*). How does effector mechanosensing change along with engineered Fc? Additionally, IgG glycosylation pattern changes in aging and diseases (*64*), which makes understanding how Fc glycosylation state regulates mechanosensing through CD16a another important direction. Fifth, the Fc region of antibodies have varied affinity to different Fc receptors and thus can engage and recruit differential pathways and immune cells to orchestrate complicated immune response. Are other Fc receptors mechanically active? Sixth, NK cells are known to have two major subsets: CD56^bright^, which is more involved in producing cytokines and immunomodulation; and CD56^dim^, which is more involved in exerting cytotoxic effector function. Stemming from the biochemical marker of CD56, on the biophysical side would we also see corresponding mechanical phenotypes of NK cells? These are all important questions in understanding the similarities and differences of cytotoxic effector function in T cells and NK cells, which will contribute to a deeper understanding of immune signaling at cell-cell interface.

In addition to the mechanistic insights and questions, our findings also imply a potential approach for antibody-based immunotherapy that is based on mechanosensing rather than affinity-based engineering. Although, at the moment, it is unclear whether CD16a-Fc forms a catch bond, we can envision that Fc variants, which can facilitate better mechanotransduction, would potentially be more potent in recruiting NK cells for ADCC. Additionally, in the TCR engineering field, it has been shown that TCR catch-bond engineering can generate superior TCR clones with high potency but low off-target properties (*60*). We can foresee that receptor mechanics-guided engineering could potentially be powerful in designing immunotherapies.

One of the limitations of this study is that our measurements were performed on glass coverslips. Although there have been speculations about the true form of actin foci at 3D cell-cell junction, there is no doubt that 2D planar glass substrate offers a great way to isolate and study the contribution from cytoskeleton. As foci structures and CD16a microclusters were previously observed on activating supported lipid bilayers (SLBs) (*65*, *66*), a promising approach would be to interrogate the actin foci dynamics with respect to receptor mechanics on SLBs as well. We suspect the actin foci could be protrusions (*67*), where CD16a on the cell membrane can quickly push and pull with the fast actin turnover under Arp2/3. Ideally, super-resolution imaging would be helpful to resolve the local mechanotransduction network through CD16a. It would also be highly desirable to track the dynamics of CD16a microcluster formation, force generation, and centralization movement with Cas-L phosphorylation on SLBs and thus resolve the kinetics of signalosome assembly in the context of force requirement and generation (*68*). At the cellular level, this study focuses on population response in Ca^2+^ and ERK phosphorylation, and it would also be desirable to include more reporters involved in different stages of activation and resolve step-wise signaling kinetics at single-cell level with respect to mechanical engagement. Additionally, we did not interrogate any potential oscillatory Ca^2+^ or ERK signaling at single-cell level, and it would be highly interesting to understand whether there is any mechanical interplay. Another limitation in this study is that we could not resolve the contribution of mechanotransduction and clustering. The field has long established the significance of avidity in antibody-mediated effector function without exploring a potential mechanosensing mechanism (*2*). While we report on CD16a mechanical activity, it is inherently difficult to fully isolate receptor mechanics from naturally formed receptor clusters. Engineered Fc multimers have demonstrated that although they induce multivalent interaction, they inhibit ADCC or ADCP processes. Perhaps an ultimate test would require the fabrication of DNA origami to limit the number of CD16a-Fc engagement in a given area, while allowing or terminating mechanical forces. On that note, studies have to carefully design the size of DNA origami platforms from the energy perspective (*69*), as they are quite rigid and might be able to provide reaction from CD16a pulling, since the CD16a-Fc forces are only ∼4 pN. Perhaps, ultimately, forces and clustering are always intertwined, and they collectively contribute to a cell’s ability for antigen discrimination: Individual receptors within the clusters could potentially withstand higher forces and facilitate signalosome assembly. Moreover, phase separation might also be contributing to forming such signaling condensates that can do more “heavy lifting” (*49*).

In conclusion, we report on a mechanosensing mechanism through the Fc receptor CD16a during NK cell activation. At a fundamental level, this research establishes CD16 as a mechanosensor, akin to TCR and BCR (*70*), which expands our understanding of Fc receptor signaling and NK cell activation, and broadens our knowledge in immunobiophysics. We expect our findings to inspire more mechanistic reevaluation of other members of FcγR family. In the future, we hope our findings can inspire fresh ideas in antibody engineering for immunotherapy, beyond the current affinity-driven approaches, and enable more effective harnessing of ADCC.

## MATERIALS AND METHODS

### Material list

The materials used in this study are listed in Table 1.

**Table 1.** Key resources used for this study.

Material name	Source, catalog no., and details (RRID)	
Antibodies		
Anti-human CD16 antibody	BioLegend 302049	AB_2783156
Biotin anti-human CD16 antibody	BioLegend 302004	AB_314204
Alexa Fluor 647 anti-human CD16 antibody	BioLegend 302020	AB_492976
Alexa Fluor 594 anti-phosphotyrosine antibody	BioLegend 309314	AB_2728249
Alexa Fluor 647 anti-LAT phospho (Tyr^171^) antibody	BioLegend 946603	AB_2927971
Alexa Fluor Plus 647 F(ab′)2-goat anti-mouse IgG (H + L) cross-adsorbed secondary antibody	Thermo Fisher Scientific A48289	AB_2896355
PE anti-human IgG1 Fc antibody	SouthernBiotech 9054-09	AB_2796628
Phospho-p130 Cas (Tyr^165^) antibody	Cell Signaling 4015	AB_2065459
Alexa Fluor 488 anti-rabbit IgG (H + L), F(ab′)2 fragment	Cell Signaling 4412S	AB_1904025
Cell culture reagents		
MEM α	Thermo Fisher Scientific	32571036
DMEM, high glucose, GlutaMAX supplement	Thermo Fisher Scientific	10566016
Freestyle 293 medium	Thermo Fisher Scientific	12338018
RPMI 1640	Corning	15-040-CV
1× DPBS	Corning	21-030-CV
Hank’s balanced salt	Sigma-Aldrich	H8264
Horse serum	Thermo Fisher Scientific	26050070
Fetal bovine serum (FBS)	Thermo Fisher Scientific	10100147
10000 U/ml penicillin and streptomycin (P/S)	Thermo Fisher Scientific	15140122
100× GlutaMAX	Thermo Fisher Scientific	35050061
Antibiotic-antimycotic (100×)	Thermo Fisher Scientific	15240062
1000× β-mercaptoethanol	Thermo Fisher Scientific	21985023
100× NEAA	Thermo Fisher Scientific	11140050
Folic acid	Sigma-Aldrich	F8758-5G
Myoinositol	Sigma-Aldrich	I7508-50G
NK Cell Isolation Kit, human	Miltenyi	130-092-657
Biological reagents		
Competent cells - DH5α	Zymo Research	T300
NK92	O. A. Aguilar (UCSF) to the Garcia laboratory	
NK92 CD16a	This study	
NK92 CD16a reporter	This study	
NK92 CD16a LifeAct	This study	
K562	ATCC	CCL-243
K562 IgG1 Fc	This study	
K562 IgG1 Fc dimer	This study	
Jurkat TCR knockout	M. Davis (Stanford) to the Garcia laboratory	
Jurkat TCR knockout NFAT reporter	This study	
Jurkat TCR knockout NFAT reporter CD16a	This study	
Lenti-X 293T cell line	Takara Bio	632180
293TB-15	NIH tetramer facility at Emory to the Garcia laboratory	
293TB-15 Fc hexamer cells	This study	
Human peripheral blood mononuclear cells (PBMCs)	Stanford Blood Center	
Chemicals		
Blocker BSA (10%) in PBS	Thermo Fisher Scientific	37525
NucBlue Live ReadyProbes Reagent	Thermo Fisher Scientific	R37605
Poly-d-lysine	Thermo Fisher Scientific	A3890401
(3-Aminopropyl)triethoxysilane	Sigma-Aldrich	440140-100ML
Pierce sulfo-NHS-acetate	Thermo Fisher Scientific	26777
EZ-Link NHS-biotin	Thermo Fisher Scientific	20217
Azidobutyric acid NHS ester	Lumiprobe	63720
Coverslips for sticky-Slides	ibidi	10812
sticky-Slide 18 Well	ibidi	T100B
CytoSoft rigidity plates	advancedbiomatrix	#5165, 5142, 5145
Cell activation cocktail (without brefeldin A)	BioLegend	423302
CK666	Cayman Chemical	29038
Latrunculin B	Cayman Chemical	10010631
Wiskostatin	Cayman Chemical	15047
Cholesterol	Thermo Fisher Scientific	A11470.18
Methyl-β-cyclodextrin (mβCD)	Thermo Fisher Scientific	J66847.06
Filipin III	Cayman Chemical	70440
Opti-MEM	Thermo Fisher Scientific	31985062
Fugene HD transfection reagent	Promega	E231A
PEG-it virus precipitation solution	System Biosciences	LV810A-1
Amicon Ultra Centrifugal Filter, 10 kDa MWCO	Sigma-Aldrich	UFC501024
SiR-actin	Cytoskeleton	CY-SC001
Particles, 0.5% (w/v), 5 μm	Spherotech	SVP-50-5
Protein A magnetic resin	GenScript	L00273
Recombinant proteins		
Recombinant human IL-2 protein	R&D systems	202-IL-010/CF
Fc hexamer	Expressed in house (*8*)	
Biotinylated human IgG1 Fc (C103S), avi tag, his tag	Acro Biosystems	IG1-H82E9-25ug
Recombinant DNA		
Codon-optimized human CD16a	IDT	
H2B-TagBFP2	Existing plasmid in laboratory	
ERKKTR-miRFP670nano3	IDT/existing plasmid in laboratory	
GCaMP6F	IDT, (*31*)	
NFAT-ZsGreen	IDT/plasmid in house	
LifeAct-mNeonGreen	IDT	
Tethered IgG1 Fc	Thermo Fisher Scientific/plasmid in house	
Tethered IgG1 Fc fragment X	Thermo Fisher Scientific/plasmid in house	
Oligonucleotides		
4 pN hairpin (IDT)	GTGAAATACCGCACAGATGCGTTTGTATAAATGTTTTTTTCATTTATACTTTAAGAGCGCCACGTAGCCCAGC	
12 pN hairpin (IDT)	GTGAAATACCGCACAGATGCGTTTGGGTTAACATCTAGATTCTATTTTTAGAATCTAGATGTTAACCCTTTAAGAGCGCCACGTAGCCCAGC	
19 pN hairpin (IDT)	GTGAAATACCGCACAGATGCGTTTCGCCGCGGGCCGGCGCGCGGTTTTCCGCGCGCCGGCCCGCGGCGTTTAAGAGCGCCACGTAGCCCAGC	
4 pN hairpin lock (IDT)	AAAAAACATTTATAC	
12 pN hairpin lock (IDT)	AGA ATC TAG ATG TTA ACC	
19 pN hairpin lock (IDT)	ACCGCGCGCCGGCCCGCGGCG	
ligand strand (IDT)	/5AmMC6/CGCATCTGTGCGGTATTTCACTTT/3Bio/	
Cy3B ligand strand (in house)	Cy3B/CGCATCTGTGCGGTATTTCACTTT/3Bio/, prepared in house, (*19*)	
BHQ2 anchor strand (IDT)	/5DBCON/TTTGCTGGGCTACGTGGCGCTCTT/3BHQ_2/	
Biotin TGT top strand (IDT)	/5BiosG/CACAGCACGGAGGCACGACAC	
12 pN TGT bottom strand (IDT)	GTGTCGTGCCTCCGTGCTGTGTTTTT/3DBCON/	
56 pN TGT bottom strand (IDT)	/5DBCON/TTTTTGTGTCGTGCCTCCGTGCTGTG	
Software and algorithms		
SnapGene	7.1.2	
FlowJo	10.10.0	
Graphpad Prism 10	10.4.2	
CellTK2	(*33*); https://github.com/sjeknic/CellTK; https://celltk.readthedocs.io/en/latest/	
ImageJ2	2.16.0. https://imagej.net/software/fiji/downloads	
Instrument		
Nikon Ti-2 microscope	Nikon Ti-E microscope with temperature and CO_2_	
Nikon CFI APO 100× Oil TIRF numerical aperture 1.49, working distance 0.12 mm	Nikon MRD01991	
RICM filter	Nikon 97271	
SCMOS camera	Andor Neo	
Fluorescence filter set	Chroma ET 572/35x,632/60 m	
Fluorescence filter set	Chroma Quad cube, DAPI/FITC/TRITC/FAR RED	

### Method details

#### Cells

NK92 cells were gifted by O. A. Aguilar (University of California, San Francisco) to the Garcia laboratory. Human CD16a F158 was transduced into NK92 cells using lentiviral particles, and the transduced cells were then sorted to establish the NK92 CD16a cell line. NK92 CD16a reporter cell line was subsequently engineered by transducing NK92 CD16a cells with lentiviral particles of H2B-TagBFP2, ERKKTR-miRFP670nano3, and GCaMP6f, followed by isolation of the transduced cells using FACS. NK92 CD16a LifeAct cell line was established by transducing NK92 CD16a cells with lentiviral particles of LifeAct-mNeonGreen and isolation of transduced cells using FACS. All NK92 cell lines were cultured in complete α-MEM [ α-MEM supplemented with 10% fetal bovine serum (FBS), 10% horse serum, and 1× NEAA, penicillin-streptomycin (P/S; 100 U/ml), 2 mM GlutaMAX, 10 mM Hepes, 20 μM folic acid, 200 μM myoinositol, 50 μM β-mercaptoethanol, and interleukin-2 (IL-2; 200 U/ml)] at 37°C with 5% CO_2_.

Jurkat TCR knockout cells were gifted by M. Davis (Stanford) to the Garcia laboratory. Jurkat TCR knockout NFAT reporter was generated by transducing Jurkat TCR knockout cells with lentiviral particles of NFAT-ZsGreen1. Jurkat TCR knockout NFAT reporter CD16a cells were generated by lentiviral transduction of human CD16a. K562 IgG1 Fc and K562 IgG1 Fc dimer cells were generated by transducing K562 cells with lentiviral particles of tethered IgG1 Fc or IgG1 Fc dimer. All Jurkat and K562 cell lines were cultured in complete RPMI 1640 medium [RPMI 1640 supplemented with 10% FBS, 2 mM GlutaMAX, 50 μM β- mercaptoethanol, and P/S (100 U/ml)] at 37°C with 5% CO_2_. Lenti-X 293T and 293TB-15 were cultured in Dulbecco’s modified Eagle’s medium (DMEM) supplemented with 10% FBS, 2 mM GlutaMAX, and 100 U P/S at 37°C with 5% CO_2_.

Human PBMCs were obtained from Stanford Blood Center. Ethical approval regarding the donors was obtained by the Stanford Blood Center (eProtocol no. 13942). The PBMCs were kept in liquid N_2_ after isolation at 10 million cells/ml. Prior to experiments, primary NK cells were purified from thawed PBMCs with MACS human NK cell isolation kit following the manufacturer’s instruction. Following purification, primary NK cells were rested for 2 hours in complete α-MEM at 37°C with 5% CO_2_ before experiments. All cell-related work was performed under biosafety protocol (APB-5682) at Stanford University.

#### Plasmids and cloning

Gene fragments of interests were purchased from IDT or Thermo Fisher Scientific using gBlock or GeneArt custom synthesis services, or cloned from previously reported plasmids from the Covert laboratory. CD16a, H2B-TagBFP2, ERKKTR-miRFP670nano3, GCaMP6f, NFAT-ZsGreen1, LifeAct-mNeonGreen, IgG1 Fc, and Fc hexamer constructs were cloned into pHR lentiviral vector using Gibson assembly. All plasmids were sequenced and verified before expressing the genes of interest.

#### Lentivirus packaging and transduction

Lenti-X 293T cells were seeded at 6.0 × 10^5^ cells per well in a six-well plate and allowed to attach overnight. On the second day, cells were transfected with 260 ng of PMD2.G, 500 ng of psPax2, and 750 ng of the plasmid of interest using FugeneHD following the manufacturer’s instructions. The medium containing plasmids were replaced with fresh complete DMEM after 16 hours, and lentivirus was harvested 48 hours later and ready to infect 10^6^ cells by titration. Lentivirus was concentrated using PEG-it virus precipitation solution and resuspended in fresh medium. For lentiviral transduction, 1 million cells were incubated in 5 ml of fresh medium containing lentivirus and polybrene (5 μg/ml) for 24 hours. Lentivirus was then removed by centrifugation, and cells were incubated in fresh medium for another 48 hours. Transduced cells were analyzed by flow cytometry 72 hours posttransduction to examine the expression of gene of interest.

#### Protein expression

293TB-15 cells were transduced with lentiviral particles to stably secrete IgG1 Fc hexamer. After transduction, 293TB-15 Fc hexamer expressing cells were cultured in freestyle 293 medium for 5 days, and the medium was collected for purification using protein A magnetic resin following the manufacturer’s protocol. The purified IgG1 Fc hexamer was desalted with Amicon filters and analyzed with native polyacrylamide gel electrophoresis (PAGE) and denaturing SDS-PAGE gels.

#### Cell sorting

Transduced cells were cultured and expanded for 5 to 7 days before FACS (Sony SH800S). Briefly, 10 to 20 million cells were spun down and resuspended in FACS buffer, and the subpopulation that expressed the genes of interest was sorted and cultured in complete medium supplemented with 1× antibiotic-antimycotic for 2 to 3 days.

#### Substrate preparation for force imaging and manipulation

DNA-functionalized substrates were prepared as described in literature (fig. S10) (*71*, *72*). Glass coverslips were washed thoroughly in ethanol and milliQ H_2_O and etched in 0.5 M KOH for 30 min. Coverslips were then washed six times with milliQ H_2_O and three times with ethanol and functionalized with 3% 3-aminopropyltriethoxysilane in ethanol. After 1 hour, coverslips were washed with ethanol six times and baked at 80°C for 20 min. The obtained amine coverslips are stored at −20°C. To further functionalize the coverslips, azide *N*-hydroxysuccinimide (NHS; 10 mg/ml in DMSO) was added to amine coverslips and allowed to react for 24 hours. Coverslips were then washed thoroughly with ethanol and incubated with sulfo-NHS acetate (10 mg/ml in DMSO) overnight. After incubation, the coverslips were washed with ethanol three times, dried, and attached to a sticky slide or bottomless adhesive microtiter plate. DNA tension probes were assembled by annealing Cy3B ligand strand, hairpin strand, and BHQ2 anchor strand (1:1:1.1) in 1× phosphate-buffered saline (PBS) at 95°C for 5 min and slowly cooling down to 20°C in 25 min using a thermal cycler. Sequence information for the oligonucleotides used for DNA tension probe technology can be found in Table 1. Azide-functionalized wells were incubated with 20 nM DNA tension probes overnight at room temperature and washed three times with 1× PBS. The wells were then passivated with 1% bovine serum albumin (BSA) for 30 min. After passivation, wells were incubated in streptavidin (10 μg/ml) for 30 min and washed three times. Biotin antiCD16 or biotin IgG1 Fc (10 μg/ml) were then added to tension probe functionalized wells for 30 min for ligand presentation, followed by three washes in 1× PBS. The wells were then ready for MTFM imaging experiments, and we estimated the density of tension probes to be around 1400/μm^2^. For TGT experiments, 5 nM annealed 12 or 56 pN TGT duplex was added to an azide-functionalized glass well instead of DNA tension probes.

#### Substrate preparation for imaging signaling dynamics

For activating substrates, wells in glass-bottom 96-well plate were coated with streptavidin (1 μg/ml) overnight at 4°C and passivated with 1% BSA in 1× PBS for 1 hour before immobilizing biotin-antiCD16 or biotin-IgG1 Fc. For nonactivating controls, wells were coated with PDL (100 μg/ml) overnight at 4°C. The wells were washed with 1× PBS immediately before use and kept in imaging buffer.

#### Imaging experiments

Microscopy imaging was performed using a Nikon Ti-E microscope with temperature and CO_2_ control for live cell imaging. The microscope is equipped with Andor Neo SCMOS camera and 10, 20, 40, and 100× objectives. Most of the signaling dynamics were acquired using the 20× objective and 3 by 3 binning in high throughput. Most of the tension data were acquired using the 100× objective and no binning. Cells were imaged in imaging buffer (Hank’s balanced salts supplemented with 10 mM Hepes and 1% FBS).

*Imaging cell-bead interactions*. Fifty microliters of streptavidin beads were blocked with 1% BSA for 30 min and then incubated with biotin IgG1 Fc (10 μg/ml) in 50 μl of 1% BSA at 4°C for 4 hours. The beads were then washed three times with 0.5 ml of 1% BSA to obtain bead-bound Fc, which was subsequently resuspended in 50 μl of imaging medium for experiments. Biotin IgG1 Fc (10 μg/ml) was used for the in-solution Fc group, and 50 μl of BSA-blocked streptavidin beads was used as empty bead group. Around 4 × 10^4^ NK92 CD16a reporter cells in 90 μl of imaging medium were seeded to each PDL-coated well in a glass-bottom 96-well plate and allowed to attach at 37°C for 20 min. As 10 μl of bead-bound Fc, in-solution Fc, or empty beads was added to reporter cells, time-lapse images were acquired in nucleus, Ca^2+^, and ERK reporter channels with 1-min intervals. The bead-bound Fc in principle should have the same amount of IgG1 Fc as the in-solution control.

*Imaging cell-cell interactions*. Around 4 × 10^4^ NK92 CD16a reporter cells were seeded in a well coated with PDL and allowed to attach for 20 min at 37°C. As K562 or K562 IgG1 Fc cells were added to NK92 CD16a reporter cells at 1:1 ratio, time-lapse images were acquired in nucleus, Ca^2+^, and ERK reporter channels with 1-min intervals. For cell stiffness manipulation, 500 mM MβCD stock solution was prepared, and 80 mM (by cholesterol) MβCD-cholesterol complex (molar ratio: 6.5/1) solution was prepared. To remove cholesterol, cells were incubated in serum-free medium containing 10 mM MβCD solution at 37°C for 30 min. To load cholesterol, cells were incubated in serum-free medium containing 500 μM (by cholesterol) MβCD-cholesterol at 37°C for 2 hours. Cells were washed twice with 1 ml of PBS at room temperature before adding to NK92 CD16a reporter cells. In parallel, cells after treatment were also stained with Filipin III (0.4 μg/ml) in 1× PBS for 30 min, washed, and imaged with 4′,6-diamidino-2-phenylindole (DAPI) cube to check cholesterol content.

*Imaging cell-substrate interaction*. To track Ca^2+^ and ERK signaling dynamics, around 4 × 10^4^ NK92 CD16a reporter cells were seeded to each activating substrate. Time-lapse images were acquired with 1-min interval in reporter channels with the 20× objective. To monitor NFAT activation, Jurkat TCR knockout CD16a NFAT reporter cells were prestained with NucBlue Live ReadyProbes Reagent to label nucleus for cell tracking and imaged with 20-min interval. For experiment groups with drug inhibition, reporter cells were pretreated with CK666, lat B, or 0.5% DMSO (control) for 20 min at 37°C before being exposed to activating substrates. To visualize actin dynamics, around 4 × 10^4^ NK92 CD16a LifeAct cells were seeded to activating substrates or PDL. Time-lapse images were acquired starting at *t* = 5 min with 10-s interval for 5 min in both F-actin and RICM channel with the 100× objective. To visualize molecular forces, NK92 CD16a cells or primary NK cells were added to MTFM substrates. Both the real-time CD16a force as well as accumulated CD16a force were imaged with the absence and presence of 1 μM lock oligonucleotide following previous report (*19*).

*Immunofluorescence staining*. Cells incubated on different substrate was fixed with 4% paraformaldehyde for 10 min and gently washed with 1× PBS, followed by permeabilization with 0.1% Triton X-100 in 1× PBS for 10 min. Cells were then washed again with 1× PBS, blocked with 5% BSA, and stained with primary antibody overnight at 4°C. On the second day, cells were washed and blocked again and stained with secondary antibody, followed by microscopy imaging.

#### Flow cytometry

*Expression of genes of interest*. Cells were stained for CD16a expression with Alexa Fluor 647–labeled antiCD16 (clone 3G8) in FACS buffer (5 μg/mL) for 30 min and washed three times before analysis on CytoFLEX flow cytometer (Beckman).

*NFAT activation*. Cells were counted, and the density was adjusted to 1 × 10^6^ cells/ml. One hundred microliters of Jurkat TCR knockout CD16a NFAT reporter cells were mixed with 100 μl of K562 IgG1 Fc or K562 IgG1 Fc dimer cells and cocultured in round-bottom 96-well plate for 16 hours at 37°C. Cells were then stained with Alexa Fluor 647–labeled antiCD16 in FACS buffer and analyzed on CytoFLEX for NFAT activation.

#### Quantification and statistical analysis

Traces of Ca^2+^ and ERK signaling dynamics were extracted from time-lapse images using a previously reported pipeline CellTK (fig. S2 and movie S1) (*33*). Briefly, nucleus segmentation was performed with U-net and thresholding to generate a nucleus mask. The nucleus mask is further expanded to generate a cytoplasm mask. The median fluorescence intensities in cytoplasm region and nucleus were obtained by applying the mask to time-lapse images and used for obtaining cytoplasm/nucleus ratio. The Ca^2+^ signal was the extracted median fluorescence intensity after applying the cytoplasm mask to GCaMP6f channel.

Images of receptor forces were processed following previously reported methods (*19*, *36*). Briefly, the fluorescence background of the quenched probes was subtracted from the force images for representative tension images. Quantitative analysis of forces, contact area, and phosphorylation in fixed samples was performed by quantifying fluorescence signaling within the ROIs of cells, and the ROIs were determined by the outline of fluorescence signal masks and/or the outline of contact area in RICM channel.

Quantitative analysis of actin foci was performed using the ImageJ Trainable Weka Segmentation tool (fig. S15). Actin foci dwell time was analyzed using the ImageJ particle tracking analysis TrackMate tool (movie S7).

Experiments were performed in triplicates. Statistical analyses were performed in GraphPad Prism and were described in each figure legend where statistical comparisons were performed, including *n* and *P* values. For dynamic signal data shown in curves, analysis and *P* values were displayed in table S1. Outliers were removed using PRISM ROUT (*Q* = 1%) function.
