# Supplementary material for: Antibody Fc receptor CD16a mediates natural killer cell activation via mechanotransduction of piconewton forces
Source: Sci Adv. 2026 Aug 28;12(35):eaeb8946. doi: 10.1126/sciadv.aeb8946 (PMC13524060; doi:10.1126/sciadv.aeb8946)
Supplement: Supplementary file 1 [file sciadv.aeb8946_sm.pdf]

Science Advances
AAAS

Supplementary Materials for

Antibody Fc receptor CD16a mediates natural killer cell activation via mechanotransduction of piconewton forces

Rong Ma *et al.*

Corresponding author: Rong Ma, rongma@stanford.edu

Sci. Adv. **12**, eaeb8946 (2026)
DOI: 10.1126/sciadv.aeb8946

The PDF file includes:

Figs. S1 to S16
Table S1
Legends for movies S1 to S10

Other Supplementary Material for this manuscript includes the following:

Movies S1 to S10

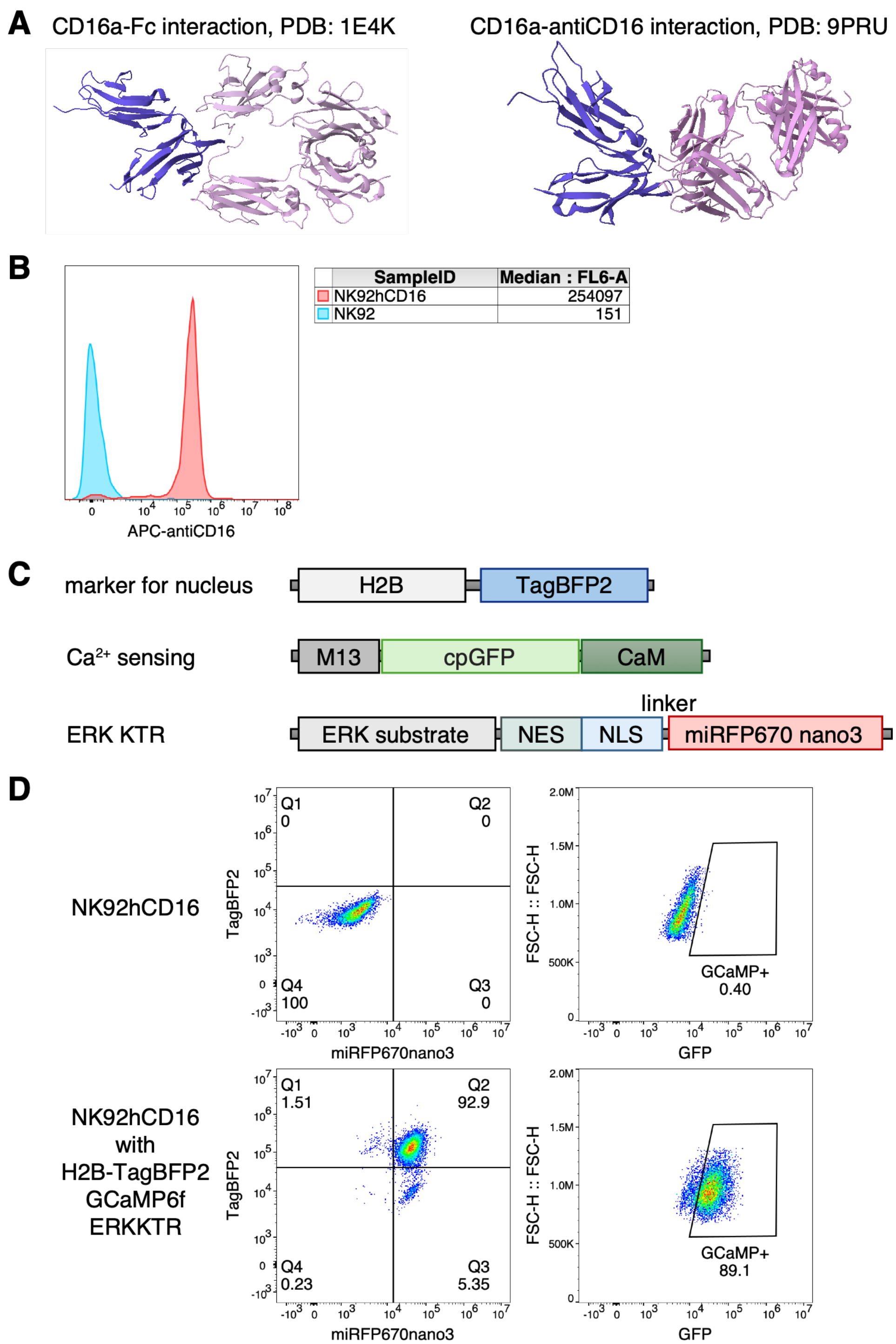

Fig. S1. CD16a interactions and the generation of the NK92 CD16a reporter cell line. (A). Interactions of CD16a and IgG1 Fc (PDB 1E4K), or CD16a and the Fab region of antiCD16 clone 3G8 (PDB 9PRU). (B) NK92 cell line was engineered to express human CD16a, and the expression was confirmed by staining with APC or Alexa647 labeled antiCD16 (clone 3G8) and analyzing with flow cytometry. (C) Constructs of fluorescent marker and reporters used to generate the NK92 CD16a reporter cell line. (D) Flow cytometry plots of NK92 CD16a cells before and after the lentiviral transduction of constructs in (C) and FACS sorting.

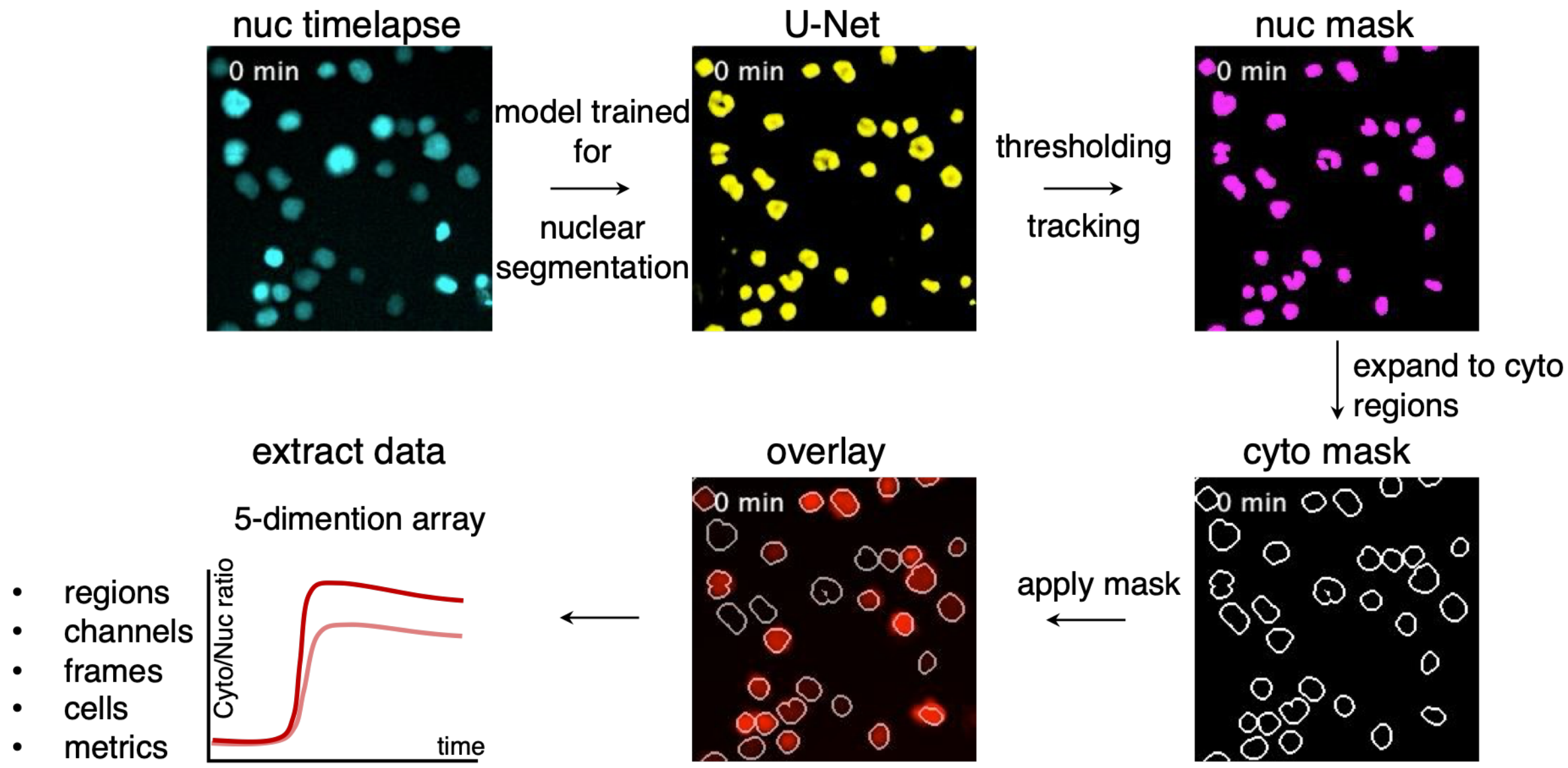

Fig. S2. Illustration of the CellTK pipeline. Cells were seeded on poly-D-lysine coated plates and allowed to attach for 20 min before imaging. Signaling traces were extracted using CellTK from timelapse images (*33*). Cell nucleus expressing H2B-TagBFP2 was captured using a DAPI cube and segmentation was performed using U-Net. After thresholding, a nuclear mask was generated and further expanded to cytoplasm regions to obtain the cytoplasm mask. Both masks were applied to raw images to obtain overlays and extract the corresponding data in a 5-dimention array, containing information of regions, channels, frames, cells, and metrics that users defined, such as Cyto/Nuc ratio (median fluorescent intensity of cytoplasm/median fluorescent intensity of nucleus of ERKKTR). The masks obtained were verified in overlayed timelapse videos (Video S1).

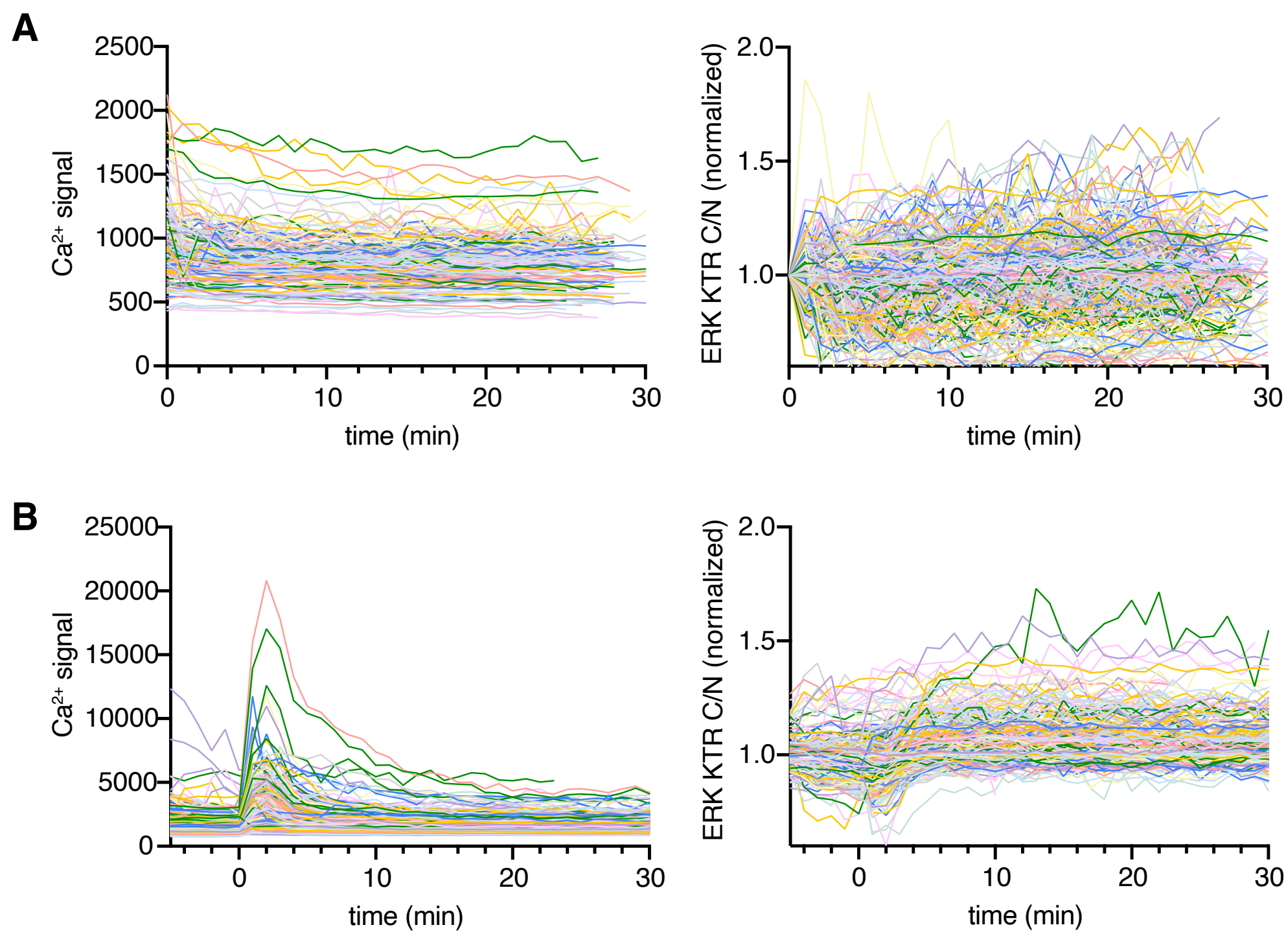

Fig. S3. Representative extracted single-cell traces of Ca^{2+} and ERK signaling activities in NK92 CD16a reporter cells. (A) Single-cell traces of Ca^{2+} and ERK signaling from ~200 cells incubated on poly-D-lysine coated glass (negative control). (B) Single-cell traces of Ca^{2+} and ERK signaling from ~200 cells incubated on poly-D-lysine coated glass with PMA ionomycin (positive control).

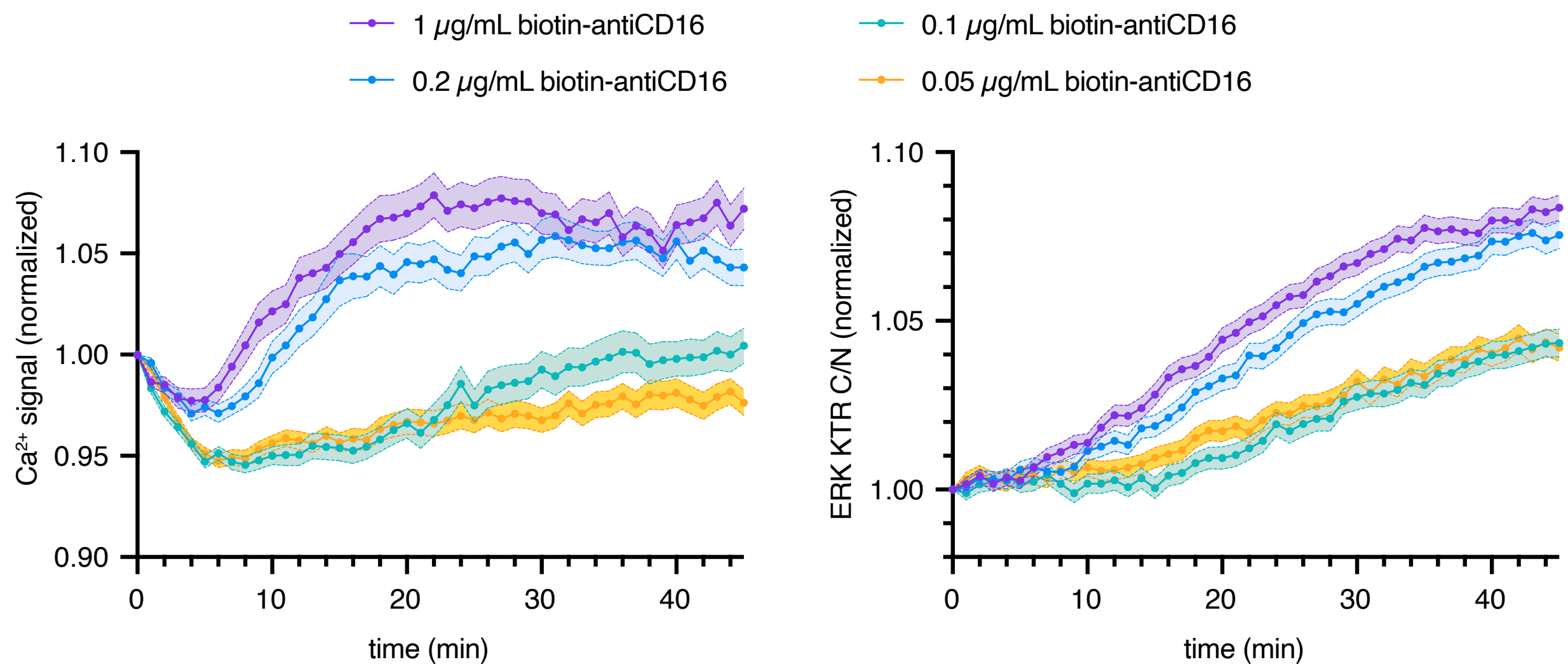

Fig. S4. NK cell activation on glass coated with different concentrations of biotin-antiCD16. Glass wells were coated with 1 μg/mL streptavidin overnight and passivated with 1% BSA for 1 h before immobilizing different concentrations of biotin-antiCD16. After washes, cells were seeded to each well and timelapse was immediately acquired with an interval of 1 min for 45 min at 37 °C with 5% CO_2. Data of each condition show mean ± SEM from >600 cells. The Ca^{2+} and ERK reporters showed clear differential activation at different antiCD16 coating concentrations.

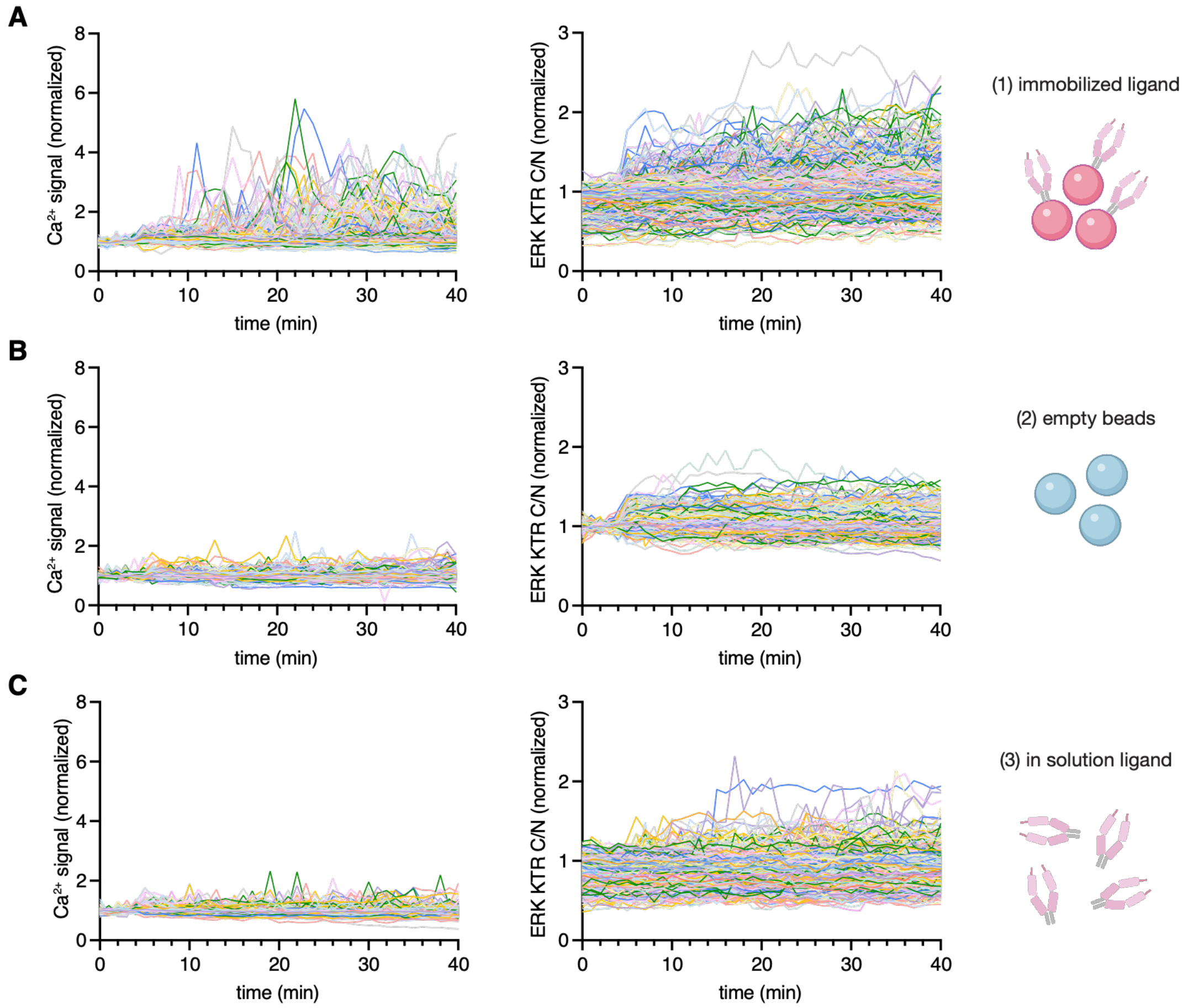

Fig. S5. Representative single-cell traces of Ca^{2+} and ERK signaling. (A) Response from NK92 CD16a reporter cells to bead-bound Fc, n = 304 cells. (B) Response from NK92 CD16a reporter cell to empty beads, n = 363 cells. (C) Response from NK92 CD16a reporter cells to soluble Fc, n = 459 cells.

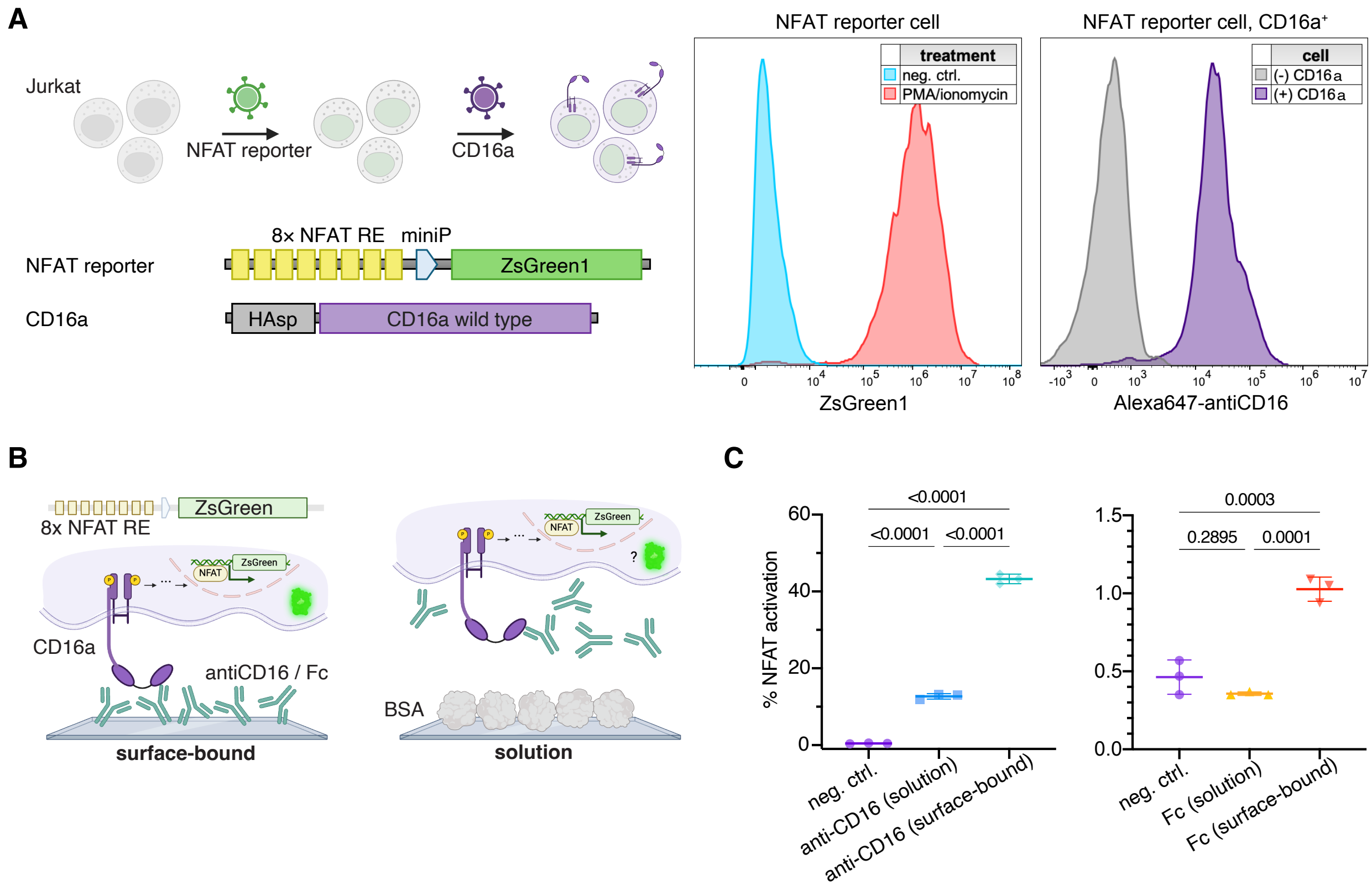

Fig. S6. NFAT activity after Fc stimulation with an engineered Jurkat CD16a NFAT reporter cell line. (A) Scheme and data showing engineering of the Jurkat CD16a NFAT reporter cell line. Jurkat TCR knockout cells were first transduced with lentiviral particles expressing NFAT reporter and then transduced again to express human CD16a. The sorted reporter cells after transduction respond well to PMA/ionomycin positive control and stained positive for CD16a using Alexa647-antiCD16. (B) The reporter cells were used to test the NFAT activation by soluble or surface-bound antiCD16 and IgG1 Fc. (C) Data shows that surface-bound antiCD16 and Fc result in higher % NFAT activation compared to antiCD16 in solution (mean ± SD, data was obtained from three replicates).

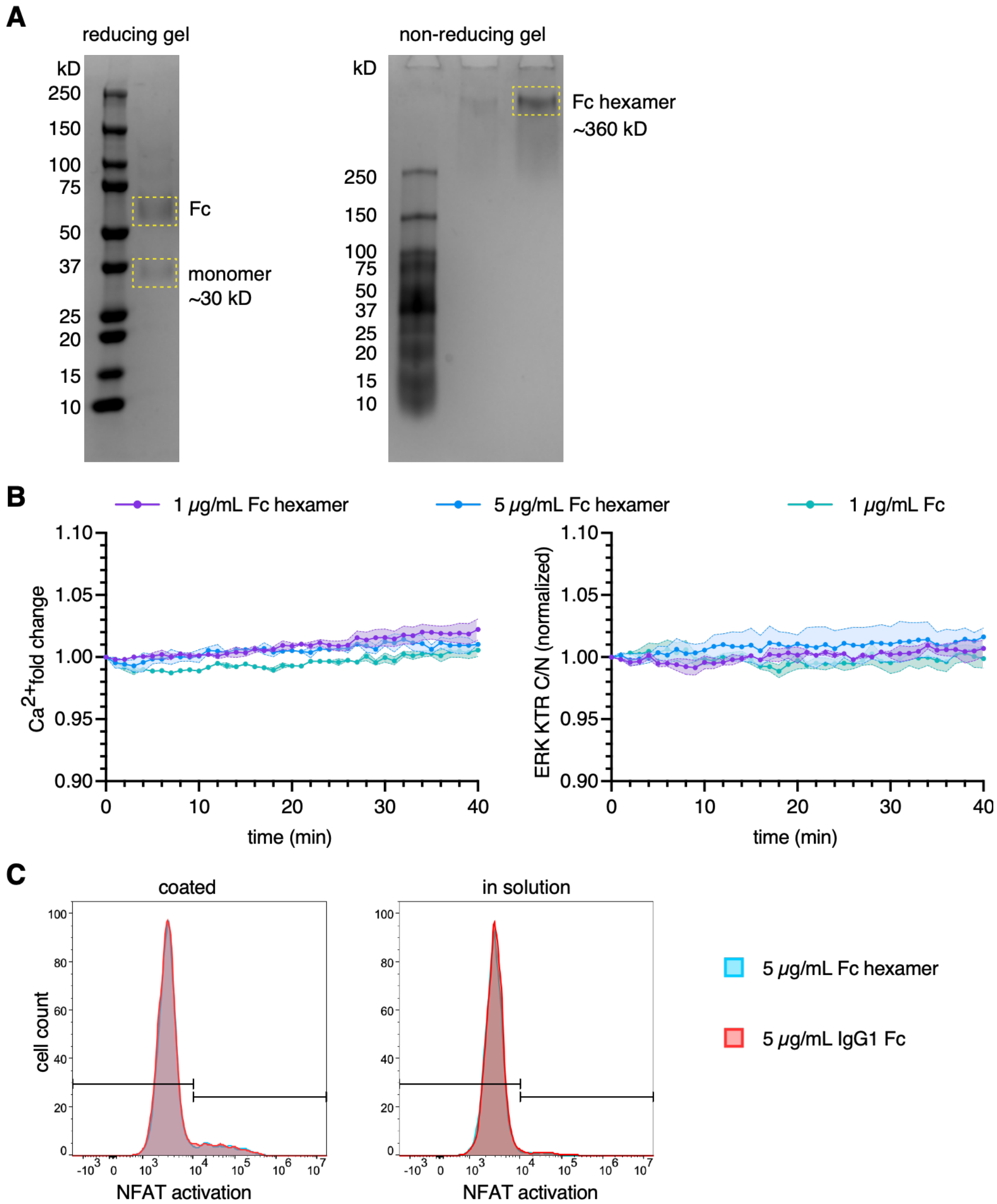

Fig. S7. Fc hexamer expression and NK cell activation. (A) Reducing and non-reducing protein gels of expressed Fc hexamer after purification showing monomers at ~ 30kDa and assembled hexamers at ~ 360 kDa. (B) Representative population response of Ca^{2+} and ERK signaling in NK92 CD16a reporter cells upon incubation with Fc or Fc hexamer in solution. Mean±SEM, n ~ 50 cells per condition in each replicate, data was obtained from three replicates. (C) Representative flow cytometry result of NFAT reporter cells after incubation with coated or in-solution Fc hexamer or IgG1 Fc.

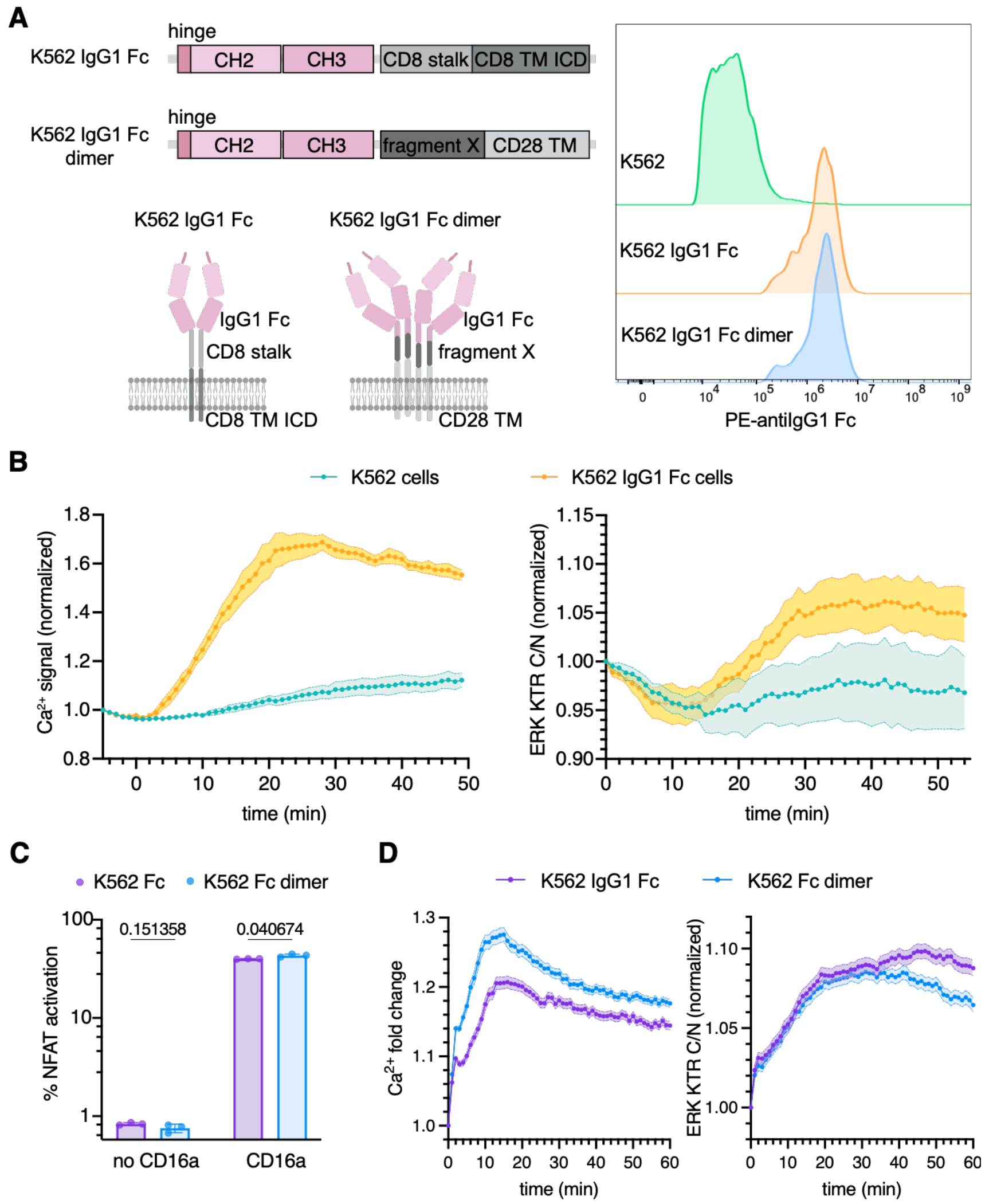

Fig. S8. Engineering K562 IgG1 Fc target cell. (A) Constructs of cell membrane-tethered Fc and Fc dimer were created and cloned into a lenti vector. K562 cells were transduced, and the expression of surface Fc was confirmed by flow cytometry using a PE-antiIgG1 Fc antibody. (B) Ca^{2+} and ERK response from NK92 CD16a reporter cells co-incubated with K562 or K562 IgG1 Fc cells. Data shows the mean±SEM, averaged from ~200-400 single cell traces in each run, and the experiment was performed in triplicate. (C) Flow cytometry data showing that two Fc expressed as one tethered dimer or four Fc expressed as 2 tethered dimers can activate CD16a with only ~3% difference in NFAT activation (mean±SEM, 3 replicates). Statistical analysis was performed with unpaired two-tail t-test. (D) Ca^{2+} and ERK response from NK92 CD16a reporter cells co-incubated with K562 IgG1 Fc versus K562 IgG1 Fc dimer cells. Data shows the mean±SEM, averaged from ~730-887 single cell traces in each run, and the experiment was performed in triplicate.

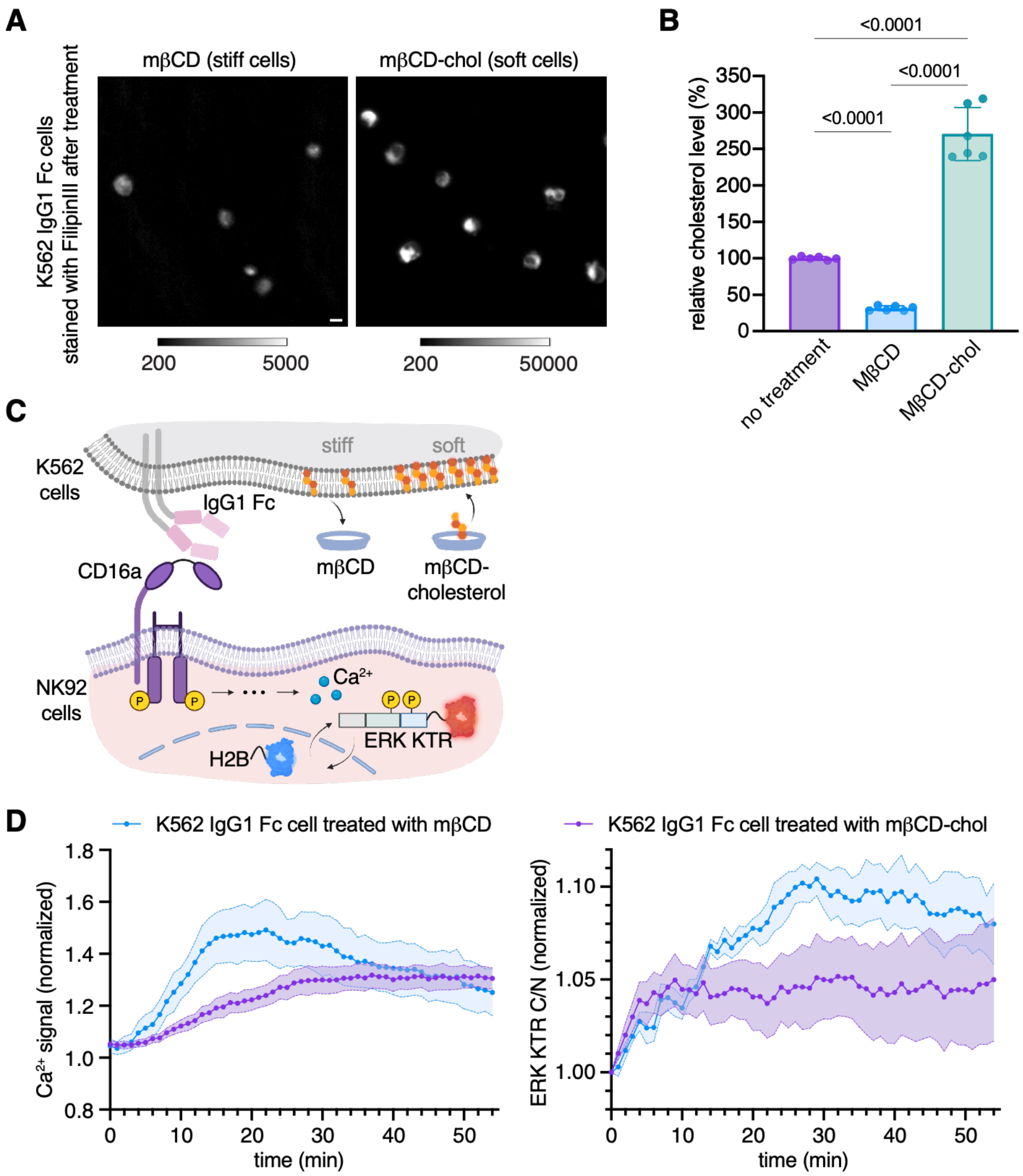

Fig. S9. Cholesterol level in K562 IgG1 Fc target cells after treatment. (A) K562 IgG1 Fc cells were treated with mβCD or mβCD-cholesterol, stained, and imaged for cholesterol content using FilipinIII. Note the calibration bars are different. Scale bar = 10 μm. (B) Quantitative analysis of relative cholesterol level upon MβCD and MβCD-chol treatment with Filipin III staining. Cells were stained with Filipin III in 1×PBS for 20 min immediately following treatment. Filipin III intensity was measured by flow cytometry. Data shows mean±SD relative cholesterol level (Filipin III intensity normalized to stained but no treatment group) from 6 independent experimental replicates. Statistical analysis was performed with one-way ANOVA and multiple comparison. (C) Scheme illustrating the experiment of co-incubating NK92 CD16 reporter cells with K562 IgG1 Fc target cells after MβCD or MβCD-cholesterol treatment. (D) Data showing that K562 IgG1 Fc target cells treated with MβCD can induce more potent Ca^{2+} flux and ERK phosphorylation in NK cells. Data (mean±SEM) collected from 3 replicates, each replicate contains single-cell traces of n~200 cells per condition.

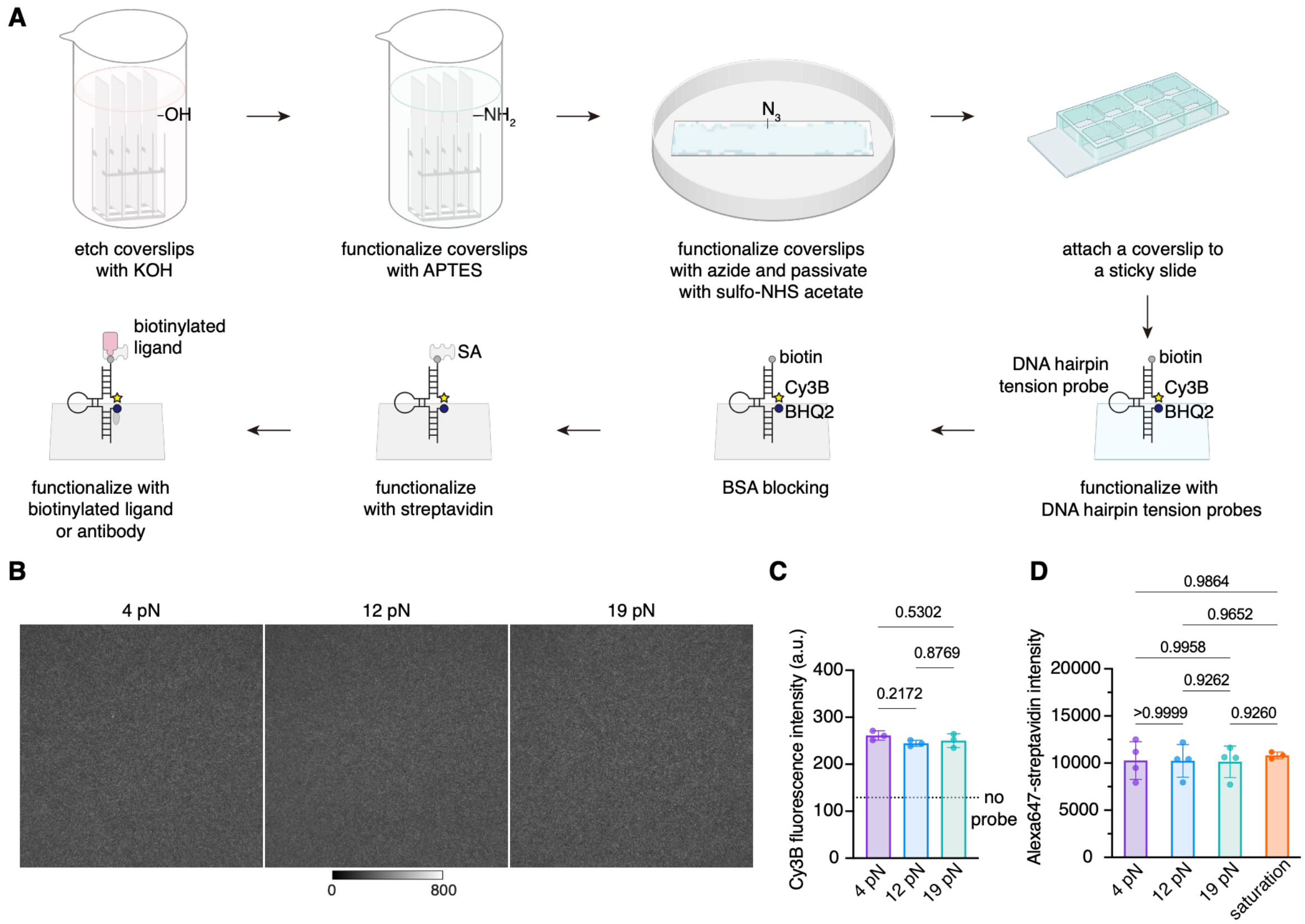

Fig. S10. Substrate preparation and characterization. (A) Scheme illustrating coverslips were first etched in KOH and functionalized with amines for conjugation with azide-NHS. The azide modified coverslips were than passivated and attached to a chamber. The coverslip is functionalized by DNA tension probes with DBCO modification and subsequently blocked by BSA solution. Immobilized biotinylated antibody or ligand was added following streptavidin incubation. (B) Representative fluorescent images of DNA tension probe substrate (Cy3B signal, collected using TRITC filter setting) with $F_{1/2}$ values of 4, 12, and 19 pN. (C) Quantitative analysis shows that the substrates of different force threshold have identical probe density. Measurement was obtained from 3 replicates, and 3 to 5 random positions from each replicate with a 100× objective. (D) Quantitative analysis shows that the substrates of probes with different force thresholds displayed identical ligand density. We incubated the substrates with 10 nM Alexa647-streptavidin (SA647) and imaged them to compare the density of biotin ligands. We also included samples that were incubated with 50 nM SA647 to ensure that we have saturated the available biotin. Measurement was obtained from 4 replicates, and ~5 random positions from each replicate with a 100× objective. Data was collected from 3 and 4 independent experimental replicates respectively, and statistical analysis was performed with paired one-way ANOVA.

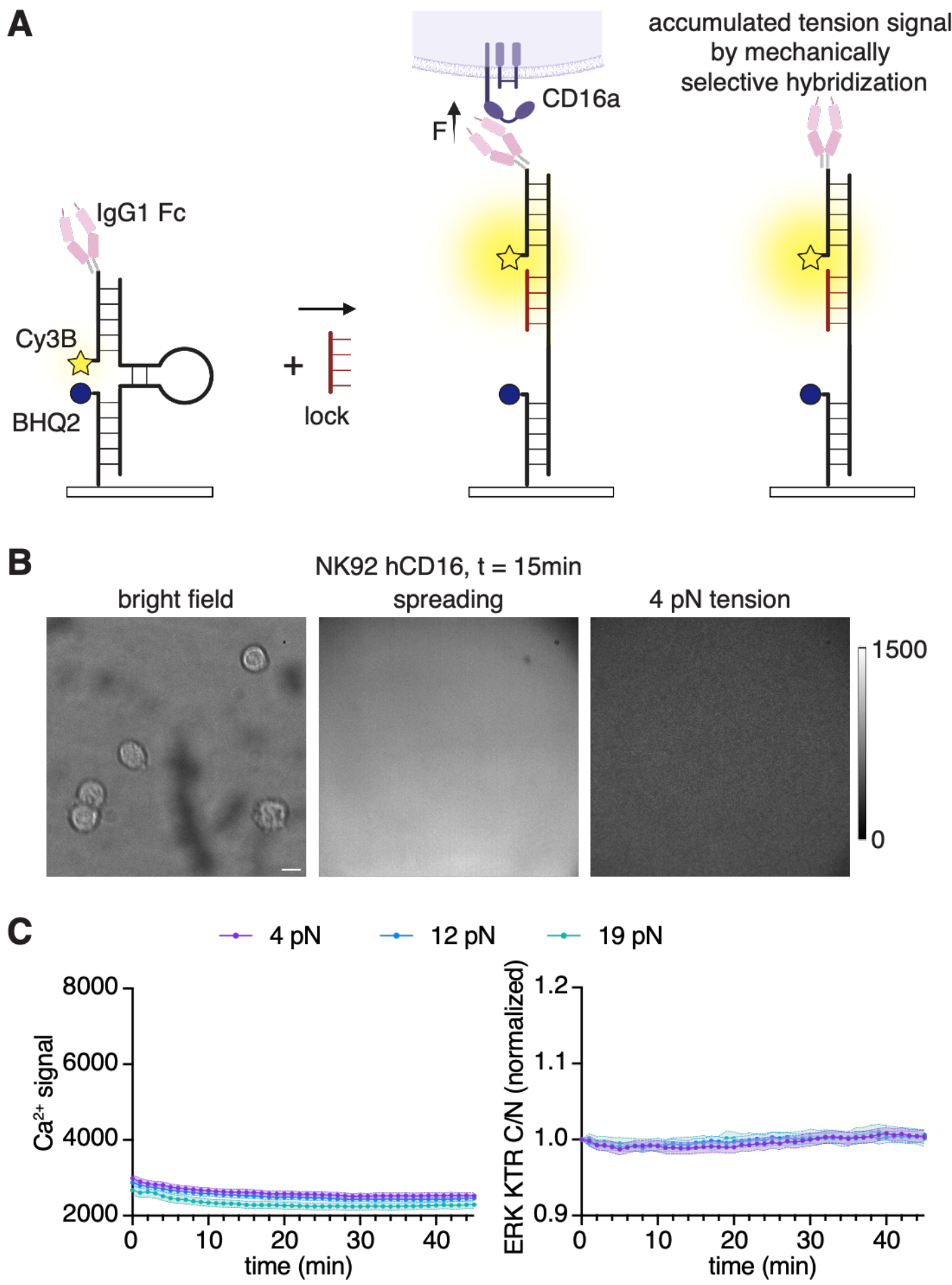

Fig. S11. Imaging on DNA tension probe substrate. (A) Schematic showing measuring accumulated force with the lock oligonucleotide (with IgG1 Fc as the ligand). Once lock is added, real-time tension signal is chemically accumulated through hybridization and therefore generates irreversible fluorescence signal upon mechanical unfolding. (B) NK92 CD16a cells were incubated on DNA probe substrates with streptavidin but without biotinylated ligands as the negative control group to confirm the specificity of CD16a forces shown in Fig. 2. No spreading or tension signal were observed with or without lock oligonucleotide. Scale bar = 5 µm. (C) Population response of NK92 CD16a reporter cells incubated on different DNA hairpin probes presenting IgG1 Fc. Experiment was performed in three replicates, data averaged (mean±SEM) from single cell traces of Ca^{2+} triggering and ERK phosphorylation, n = 319 to 765 cells per condition. While there were a few cells that were activated after engaging the Fc tension probe in each run, these activation signals were buried in the averaged population response. The ligand density on chemically modified probe substrate was not as high as coated substrates, which could contribute to the difficulties to visualize population-level signal on Fc probes.

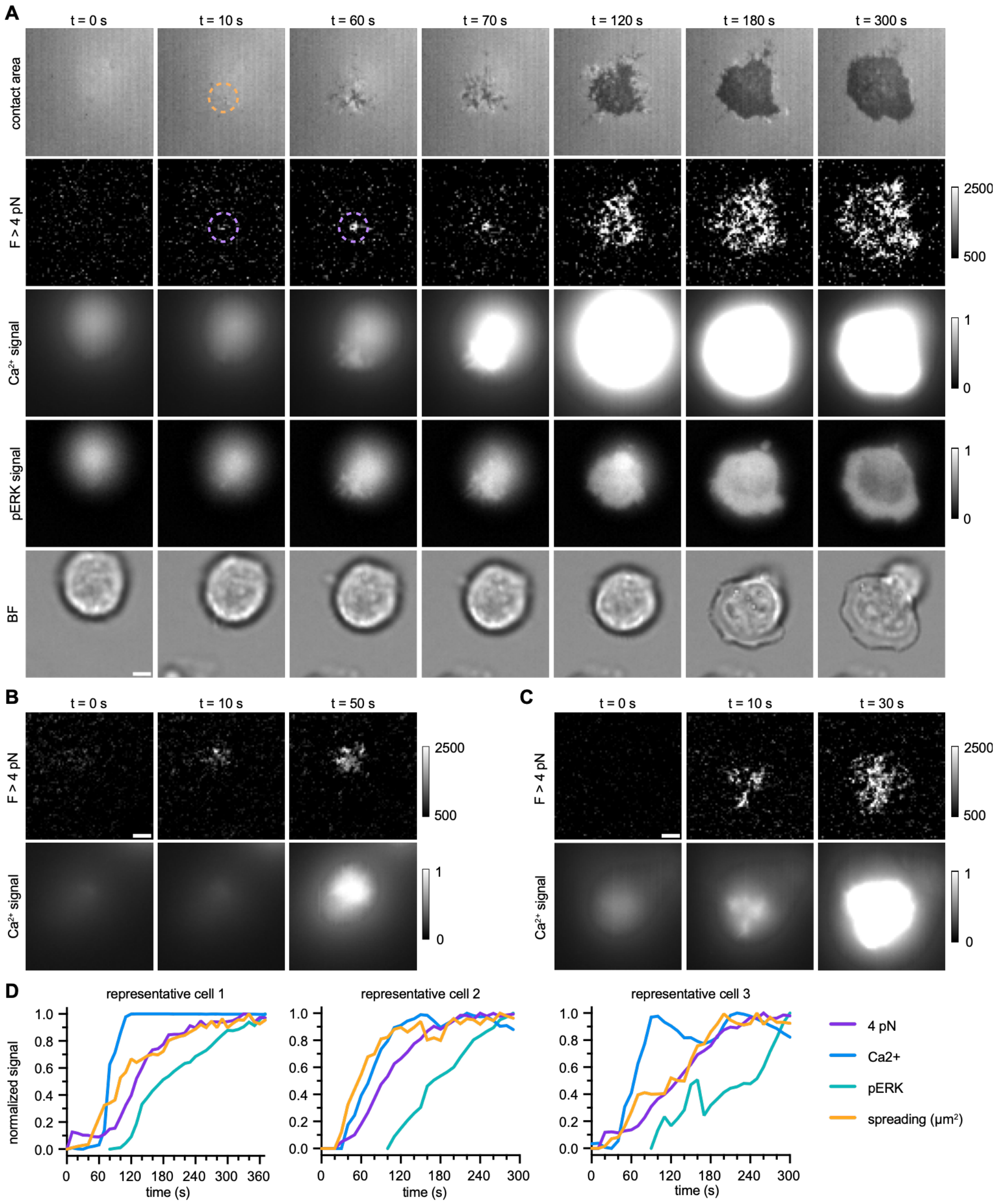

Fig. S12. Sequential events during early activation. (A) Representative images of the sequence of events for an NK92 CD16a reporter cell during activation. Once the cell contacted the ligand, force transmission was immediately observed through CD16a, followed by Ca^{2+} triggering, then ERK phosphorylation. Images shown are before contacting (0 s), at first contact (10 s), just before Ca^{2+} triggering (60 s), at first capture of Ca^{2+} triggering (70 s), at highest Ca^{2+} signal (120 s), during ERK signaling (180 s), and reaching highest ERK phosphorylation (300 s). Scale bar = 10 µm. (B) and (C) The sequence of events during activation of force-Ca^{2+}-pERK was consistently observed, but with varying time for each event in different cells. For example, the cell in panel (B) showed force transmission starting from 10 s, and Ca^{2+} did not respond until t = 50 s, while the cell in panel (C) showed force transmission at t = 10 s accompanied by minimal Ca^{2+} signal and full Ca^{2+} triggering at t = 30 s. (D) Kinetic analysis of representative cells.

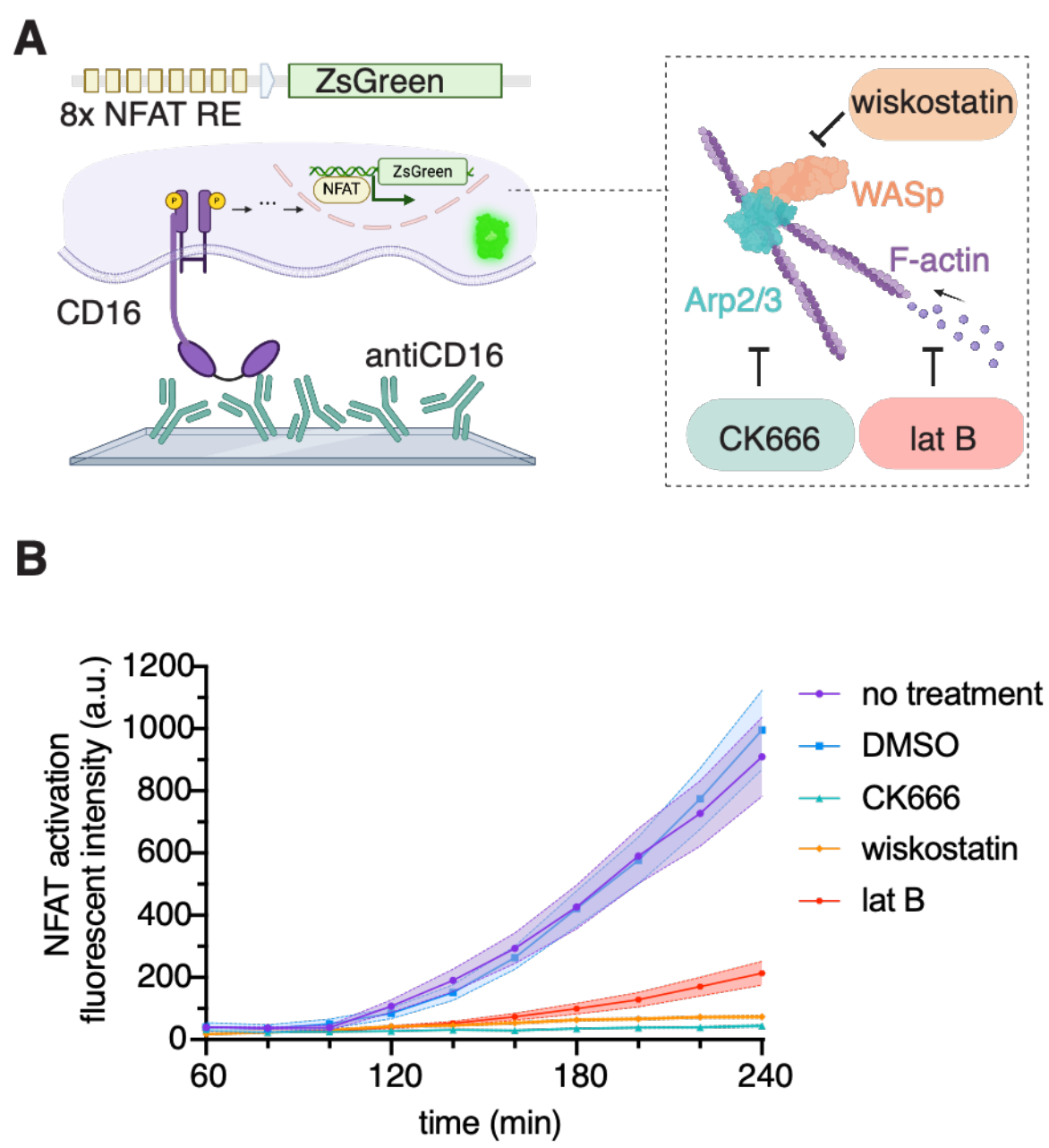

Fig. S13. NFAT activation was inhibited by CK666, wiskostatin, and lat B treatment in Jurkat CD16a NFAT reporter cells. (A) Scheme showing Jurkat CD16a NFAT reporter cells were pretreated with DMSO, CK666, wiskostatin, or lat B at 37 °C for 20 min, and then plated on antiCD16 coated wells for activation. (B) Single cells were tracked with a live nuclear stain during imaging and activation in NFAT was measured by ZsGreen1 intensity. Data show mean±SEM from three replicates, n = 691, 954, 2006, 446, and 1653 cells for no treatment, DMSO, CK666, wiskostatin, and lat B group respectively. Cytoskeleton inhibition significantly dampens NK activation through CD16a.

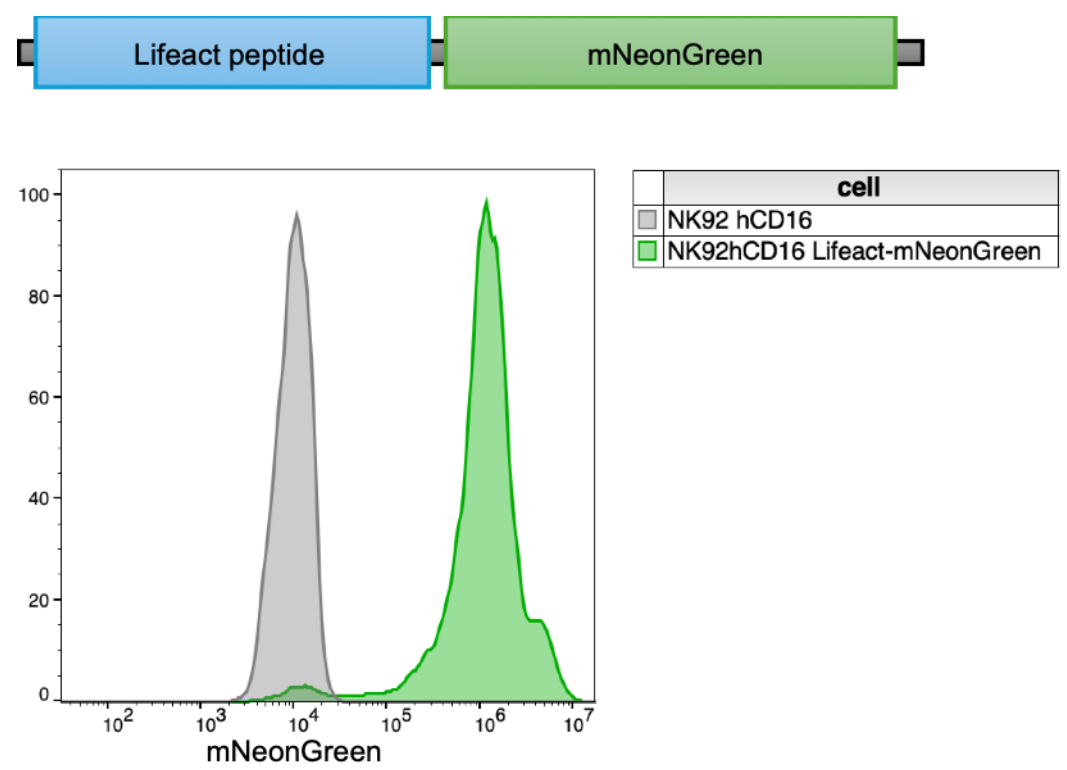

Fig. S14. NK92 CD16a expressing F-actin marker. NK92 CD16a cells were transduced with lentiviral particles to express the Lifeact-mNeonGreen. Lifeact is a short peptide MGVADLIKKFESISKEE and binds to filamentous actin, which can be used for imaging F-actin dynamics. The stably expressing cells were sorted by FACS and cultured for experiments where visualization of F-actin dynamics was required.

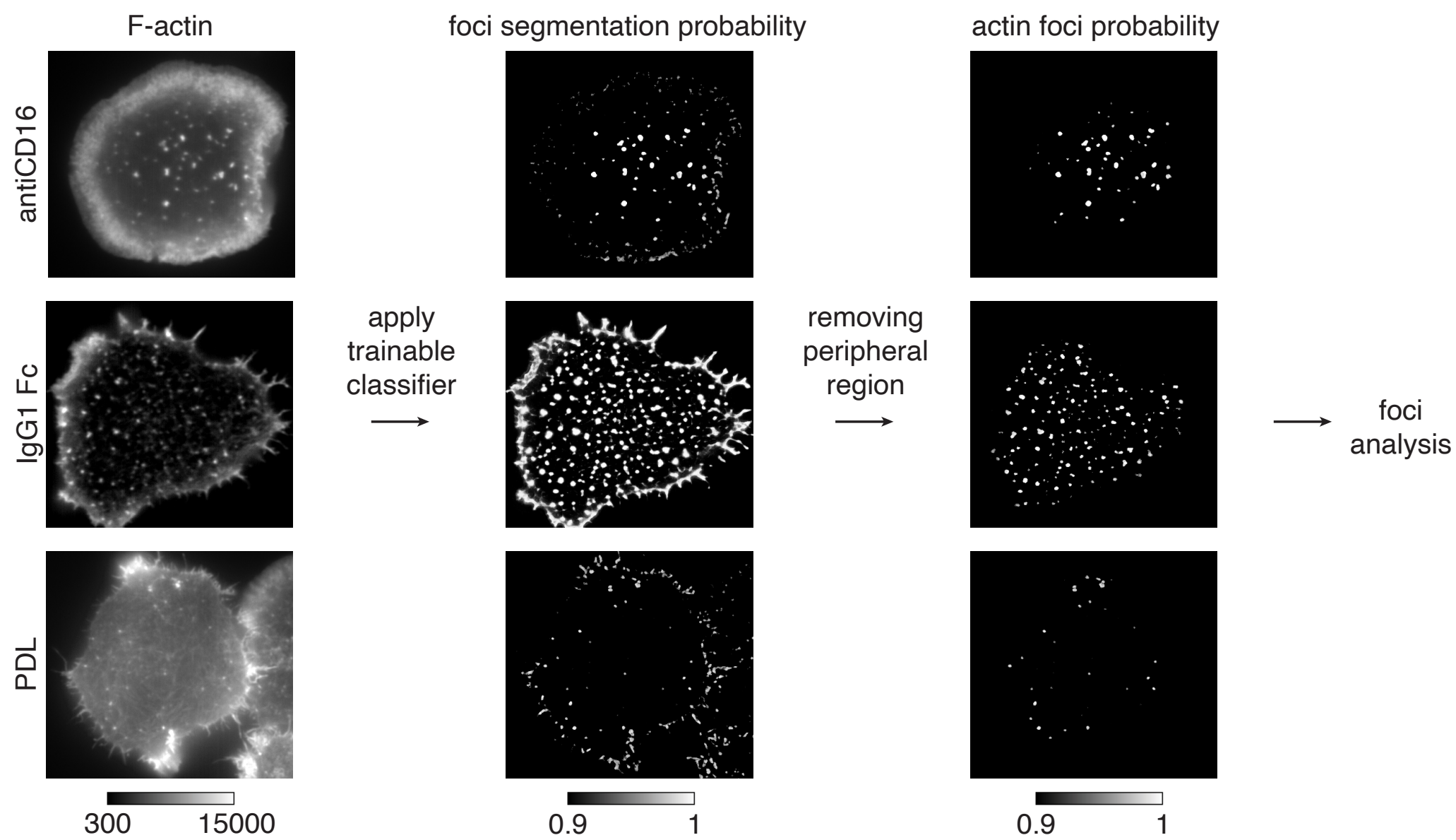

Fig. S15. Actin foci quantification in NK92 CD16a lifeact-mNeonGreen cells. Images of cells were collected in lifeact-mNeonGreen channel, and the actin foci were segmented using a trainable classifier. The classifier was then generated using Image J Trainable Weka Segmentation tool. The classifier was applied to images of cells to obtain segmented actin foci with > 0.9 probability for quantitative analysis.

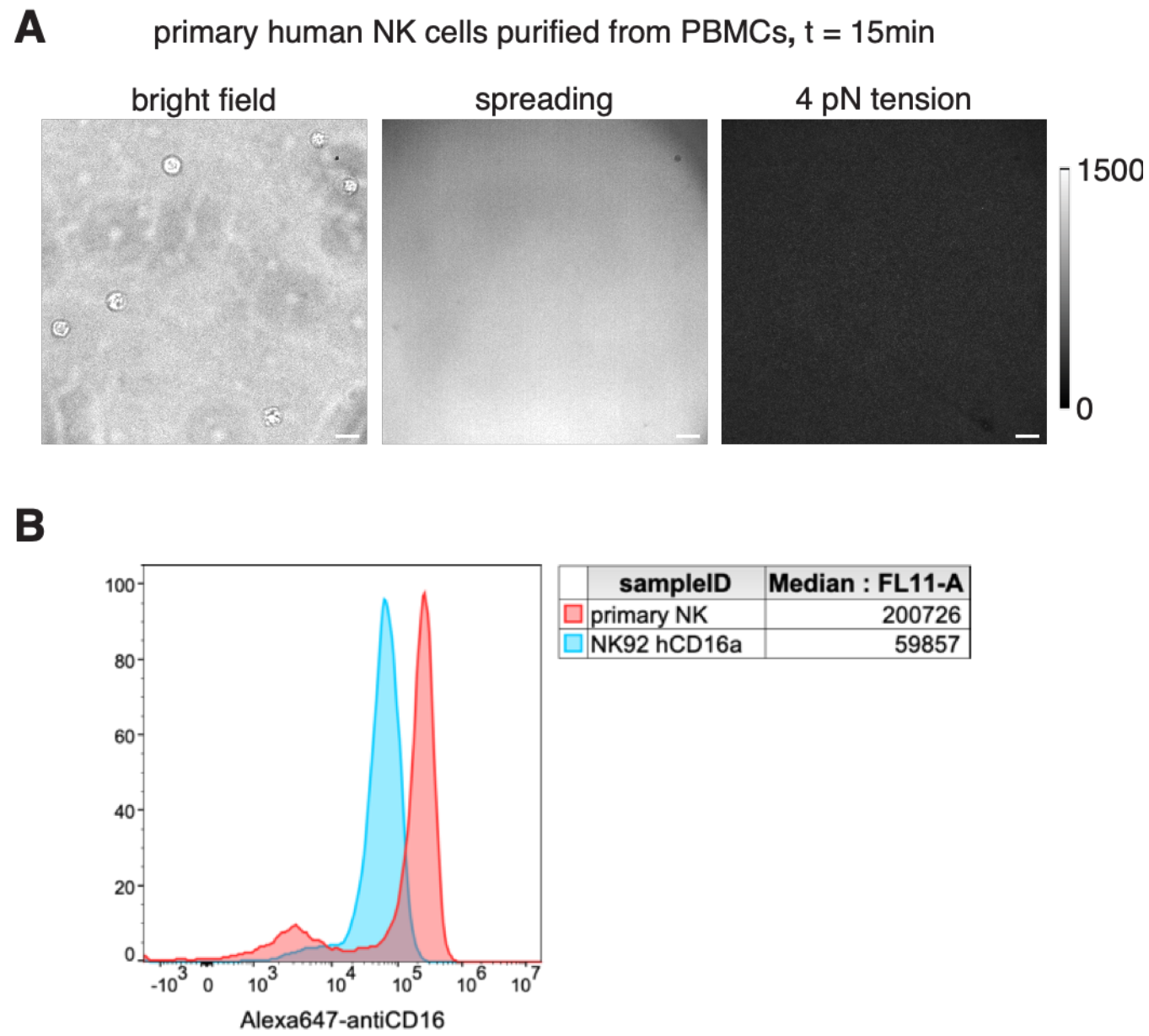

Fig. S16. Control and CD16a expression in primary human NK cells. (A) Primary NK cells were incubated on DNA probe substrates with streptavidin but no biotinylated ligands. Bright field images indicate the presence of the cells, but no contact or tension was observed in RICM and tension channels after 15 min of incubation. Scale bar = 5 µm. (B) CD16a expression level comparison between primary NK and NK92 hCD16a cells. Cells were stained in FACS buffer for 30 min at 4 ºC in dark with 10 µg/mL Alexa647-antiCD16 and washed three times before flow cytometry measurements.

statistical analysis	figure	multiple comparisons test	p value
**p values in main figures**			
2-way ANOVA, Tukey's multiple comparison, main column effect	Fig. 1G Ca	bead-bound Fc vs. in solution Fc	<0.0001
		bead-bound Fc vs. beads	<0.0001
		in solution Fc vs. beads	0.9735
	Fig. 1G ERK	bead-bound Fc vs. in solution Fc	<0.0001
		bead-bound Fc vs. beads	<0.0001
		in solution Fc vs. beads	0.0864
2-way ANOVA, Tukey's multiple comparison, main column effect	Fig. 2G, Ca2+	4 pN vs. 12 pN	<0.0001
		4 pN vs. 19 pN	<0.0001
		12 pN vs. 19 pN	<0.0001
	Fig. 2G, ERK	4 pN vs. 12 pN	<0.0001
		4 pN vs. 19 pN	<0.0001
		12 pN vs. 19 pN	<0.0001
2-way ANOVA, Tukey's multiple comparison, main column effect	Fig. 3B	no treatment vs. DMSO	<0.0001
		no treatment vs. CK666	<0.0001
		no treatment vs. wiskostatin	<0.0001
		no treatment vs. lat B	<0.0001
		no treatment vs. neg. ctrl. (poly-D-lysine)	<0.0001
		DMSO vs. CK666	<0.0001
		DMSO vs. wiskostatin	<0.0001
		DMSO vs. lat B	<0.0001
		DMSO vs. neg. ctrl. (poly-D-lysine)	<0.0001
		CK666 vs. wiskostatin	<0.0001
		CK666 vs. lat B	<0.0001
		CK666 vs. neg. ctrl. (poly-D-lysine)	0.6897
		wiskostatin vs. lat B	0.9586
		wiskostatin vs. neg. ctrl. (poly-D-lysine)	<0.0001
		lat B vs. neg. ctrl. (poly-D-lysine)	<0.0001
**p values in SI figures**			
2-way ANOVA, Tukey's multiple comparison, main column effect	Fig. S4	1 μg/mL biotin-antiCD16 vs. 0.2 μg/mL biotin-antiCD16	<0.0001
		1 μg/mL biotin-antiCD16 vs. 0.1 μg/mL biotin-antiCD16	<0.0001
		1 μg/mL biotin-antiCD16 vs. 0.05 μg/mL biotin-antiCD16	<0.0001
		0.2 μg/mL biotin-antiCD16 vs. 0.1 μg/mL biotin-antiCD16	<0.0001
		0.2 μg/mL biotin-antiCD16 vs. 0.05 μg/mL biotin-antiCD16	<0.0001
		0.1 μg/mL biotin-antiCD16 vs. 0.05 μg/mL biotin-antiCD16	<0.0001
2-way ANOVA, Tukey's multiple comparison, main column effect	Fig. S7B	F074_1ugmL vs. F074_5ugmL	0.0001
		F074_1ugmL vs. Fc_1ugmL	<0.0001
		F074_5ugmL vs. Fc_1ugmL	<0.0001
		F074_1ugmL vs. F074_5ugmL	<0.0001
		F074_1ugmL vs. Fc_1ugmL	0.5299
		F074_5ugmL vs. Fc_1ugmL	<0.0001
unpaired two-tailed t test of curve AUC	Fig. S8B	AUC_Ca	<0.0001
		AUC_ERK	0.2373
	Fig. S8C	AUC_Ca	0.0867
		AUC_ERK	0.5498
unpaired two-tailed t test of curve AUC	Fig. S9D	AUC_Ca	0.0008
		AUC_ERK	0.001
2-way ANOVA, Tukey's multiple comparison, main column effect	Fig. S11C, Ca	4 pN vs. 12 pN	<0.0001
		4 pN vs. 19 pN	0.0002
		12 pN vs. 19 pN	0.977
	Fig. S11C, ERK	4 pN vs. 12 pN	0.0382
		4 pN vs. 19 pN	0.0006
		12 pN vs. 19 pN	0.1743
2-way ANOVA, Tukey's multiple comparison, main column effect	Fig. S13	no treatment vs. DMSO	0.9997
		no treatment vs. CK666	<0.0001
		no treatment vs. wiskostatin	<0.0001
		no treatment vs. lat B	<0.0001
		DMSO vs. CK666	<0.0001
		DMSO vs. wiskostatin	<0.0001
		DMSO vs. lat B	<0.0001
		CK666 vs. wiskostatin	0.9892
		CK666 vs. lat B	<0.0001
		wiskostatin vs. lat B	0.024

Table S1. Statistical analysis and p values of dynamic signaling data.

Video S1. Single-cell signaling dynamics tracking with CellTK

Video S2. Ca^{2+} and ERK signaling when NK cells interact with stiff or soft target cells

Video S3. NK92 CD16a cells transmitting forces above 4 pN against human IgG1 Fc

Video S4. NK92 CD16a reporter cell contacting antiCD16, producing above 4 pN force, followed by Ca^{2+} triggering and ERK phosphorylation

Video S5. Activation of NK92 CD16a reporter cells on 4, 12 , and 19 pN hairpin tension probes

Video S6. F-actin foci dynamics on different substrates. Left to right: poly-D-lysine, antiCD16, and human IgG1 Fc.

Video S7. TrackMate Capture of F-actin foci on different substrates.

Video S8. CD16a force transmission and F-actin dynamics

Video S9. Primary NK cells generating CD16a forces greater than 4 pN against IgG1 Fc

Video S10. Primary NK cells generating CD16a forces greater than 4, 12, or 19 pN against antiCD16
